# Assessing SNP genotyping of noninvasively collected wildlife samples using microfluidic arrays

**DOI:** 10.1038/s41598-017-10647-w

**Published:** 2017-09-07

**Authors:** Alina von Thaden, Berardino Cocchiararo, Anne Jarausch, Hannah Jüngling, Alexandros A. Karamanlidis, Annika Tiesmeyer, Carsten Nowak, Violeta Muñoz-Fuentes

**Affiliations:** 1 Conservation Genetics Group, Senckenberg Research Institute and Natural History Museum Frankfurt, Clamecystraße 12, 63571 Gelnhausen, Germany; 2 Department of Ecology and Evolution, Johann Wolfgang Goethe-University, Biologicum, Max-von-Laue-Straße 13, 60438 Frankfurt am Main, Germany; 3 ARCTUROS, Civil Society for the Protection and Management of Wildlife and the Natural Environment, Aetos, GR-53075 Florina, Greece; 4 Department of Ecology and Natural Resource Management, Norwegian University of Life Sciences, 1432 Ås, Norway; 5 Present Address: European Molecular Biology Laboratory, European Bioinformatics Institute, Wellcome Trust Genome Campus, Hinxton, Cambridge, CB10 1SD UK

## Abstract

Noninvasively collected samples are a common source of DNA in wildlife genetic studies. Currently, single nucleotide polymorphism (SNP) genotyping using microfluidic arrays is emerging as an easy-to-use and cost-effective methodology. Here we assessed the performance of microfluidic SNP arrays in genotyping noninvasive samples from grey wolves, European wildcats and brown bears, and we compared results with traditional microsatellite genotyping. We successfully SNP-genotyped 87%, 80% and 97% of the wolf, cat and bear samples, respectively. Genotype recovery was higher based on SNPs, while both marker types identified the same individuals and provided almost identical estimates of pairwise differentiation. We found that samples for which all SNP loci were scored had no disagreements across the three replicates (except one locus in a wolf sample). Thus, we argue that call rate (amplification success) can be used as a proxy for genotype quality, allowing the reduction of replication effort when call rate is high. Furthermore, we used cycle threshold values of real-time PCR to guide the choice of protocols for SNP amplification. Finally, we provide general guidelines for successful SNP genotyping of degraded DNA using microfluidic technology.

## Introduction

Noninvasively collected samples play an important role in wildlife genetic studies. The collection of animal residues, such as hair, scats, saliva and feathers, enables the study of wild and elusive species that are otherwise difficult to sample, whilst minimizing disturbance to the animals and their habitats^1–5^. However, the extraction and subsequent analyses of DNA from noninvasively collected samples present considerable challenges^6–8^. The recovered DNA is often degraded and quantities are usually low, resulting in decreased amplification success and increased processing efforts to obtain genotypes of sufficient quality^9–11^. Co-extracted PCR inhibitors, especially from scat samples, may further complicate the generation of reliable genotype data.

In the past years, genome-wide single nucleotide polymorphisms (SNPs) have become increasingly popular as a marker of choice in population genetic studies^12–17^. SNPs are valued as an efficient and cost-effective addition to the toolkit of genetic markers^5, 18^. Assessing population structure or hybridization, individual identification, as well as sex, parentage and relatedness determination are examples of key applications in population genetic studies for which SNP loci have been selected^19^. Consequently, SNP marker panels have been used in a number of conservation genetic studies to date (e.g. wild guppies, *Poecilia reticulata* ^14^; mountain ponies, *Equus ferus caballus* ^20^; Atlantic salmon, *Salmo salar* ^21^). However, the technology has not reached routine genetic monitoring for many species, which would be a prerequisite for joint cross-laboratory conservation efforts^5^.

SNPs have considerable advantages compared to the more traditionally applied microsatellite markers^5, 19, 22^. Unlike microsatellite scores, SNP data do not require calibration across different laboratories and therefore the data can be readily compared. Also, the mutation modes are better known for SNPs, and thus ambiguities due to null alleles or variable mutation patterns are less frequent than in microsatellite studies^19^. Genotype calls are rather straight-forward and the bi-allelic nature of the markers leaves little room for subjective scoring decisions. Using microfluidic platforms, genotype data for up to 192 samples can be generated within a few hours, allowing for high-throughput analyses. With the advent of next generation sequencing (NGS) technologies, panels of SNPs can easily be generated (e.g., refs 15 and 23–25), including for non-model organisms^26–28^. Importantly, selection of markers might lead to ascertainment bias in the panel composition, e.g., if loci with high *F*_ST_ values have been selected to distinguish individuals in a certain population only^29^. These kinds of implications need to be considered when assembling or adopting SNP marker panels, as is also the case for other marker systems, such as microsatellites^30–32^.

The requirement of samples with high DNA quantity and quality has complicated the implementation of SNP markers in wildlife monitoring for a long time^33, 34^. Recently emerged microfluidic platforms, however, rely on the amplification of very short amplicons (typically less than 120 bp). Thus they might be particularly suitable for the amplification of fragmented DNA, such as DNA extracted from noninvasively collected or historical material.

In this study, we address the issue of how to produce, assess and apply SNP data to samples with low DNA quantity and quality using microfluidic platforms. We focus on how reliable SNP data can be generated from this type of samples, and how the data can be assessed in order to minimize replication. Finally, we compare the success in obtaining SNP- versus microsatellite-based genotypes.

To answer these questions, we generated SNP data for hair and scat samples of grey wolves, European wildcats and brown bears. We assessed SNP genotyping performance across replicates and in relation to real-time PCR (RT-PCR) measurements. In addition, we compared the performance of SNP and microsatellite genotyping data generated from the same samples. Finally, based on our results, we provide general SNP genotyping guidelines for noninvasive samples that may be applicable to other projects aiming to use microfluidic SNP genotyping arrays.

## Results

### SNP genotyping performance

**Figure 1:**
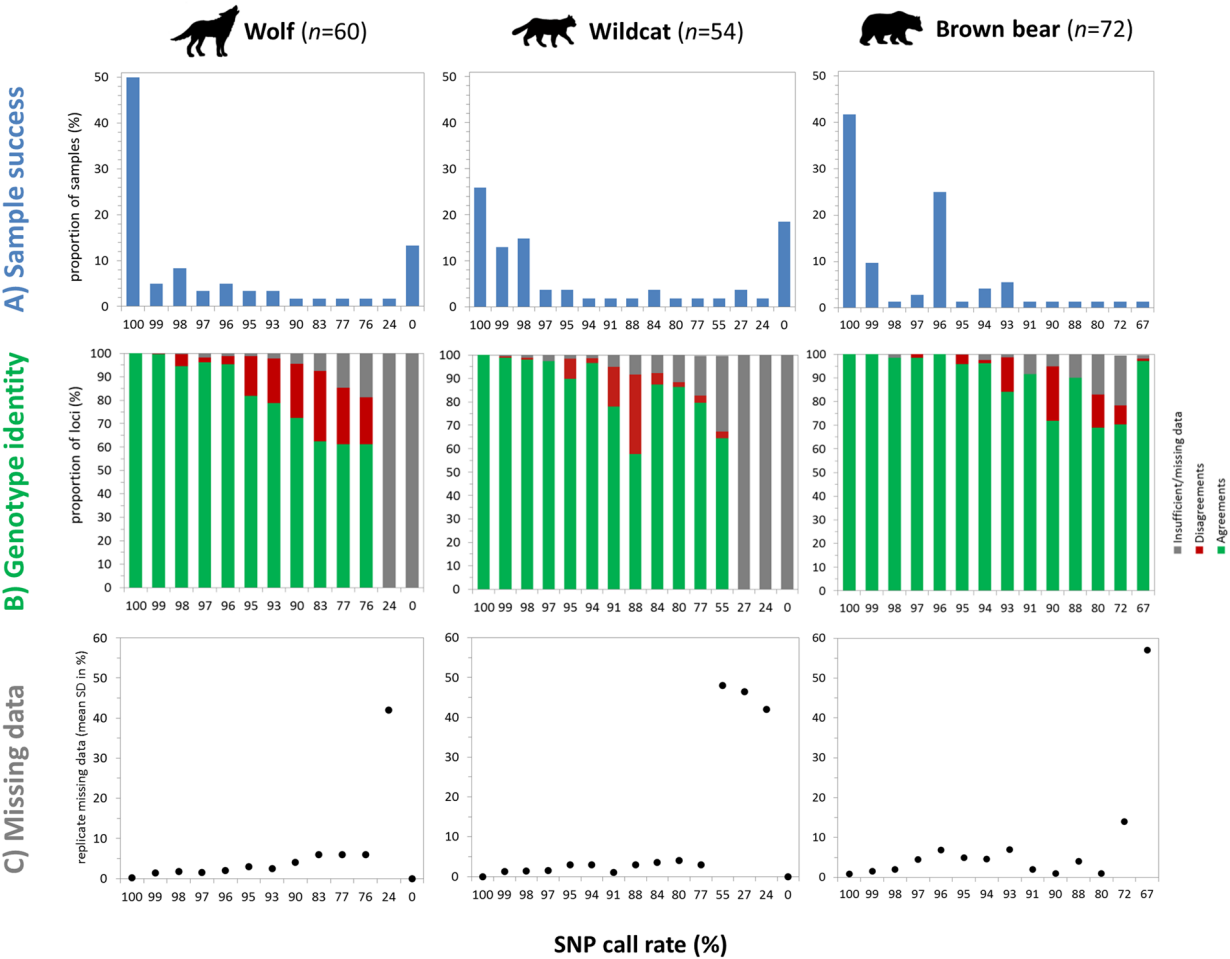
Assessment of sample performance and genotype consistency. Each sample was genotyped three times. Loci that failed to amplify in ≥70% of the reactions were removed. The SNP call rate indicates the proportion of scored loci per sample, averaged across the three genotypes. (**A**) Overview of sample success. (**B**) Proportion of loci with no mismatches (across two or three replicates, green) or with disagreements (at least one replicate, red) or with insufficient/missing data (two or three replicates failed, grey). (**C**) Variability in missing data measured as the mean of the standard deviations for the percentage of missing data averaged across the three replicates.

We generated SNP genotypes for noninvasively collected samples based on three PCR replicates for three carnivore species and investigated genotyping performance using available panels of 96 SNP loci^35–37^. We removed loci that failed to amplify in ≥70% of the reactions (16 for wolves, 37 for cats and 25 for bears) to minimize missing data, and calculated the percentage of samples that got called for 0–100% of the remaining loci, averaged over the three replicates (Fig. 1A). Call rates of 100% were obtained for 50% of the wolf samples (scats), 26% of the cat samples (hair) and 42% of the bear samples (hair).

Our results were similar across the three species. As SNP call rates decreased, disagreements across sample replicates increased (i.e., at least one disagreement per locus out of two or three successful PCR replicates; Fig. 1B), and so did the number of loci with insufficient/missing data (only one PCR replicate amplified or no amplification in all replicates). Notably, samples without missing data (call rate of 100%) had no disagreements, except for one locus in a wolf sample. For samples with an average call rate of <70% we did not attempt to obtain a consensus genotype. We also noted that the variability in rates of missing data across the three replicates increased as a proportion of decreasing average SNP call rate (Fig. 1C). For samples with SNP call rates <70%, the mean SD of missing data increased to more than 40% across replicates.

### Comparison of SNP and microsatellite amplification against cycle threshold values of real-time PCR

**Figure 2:**
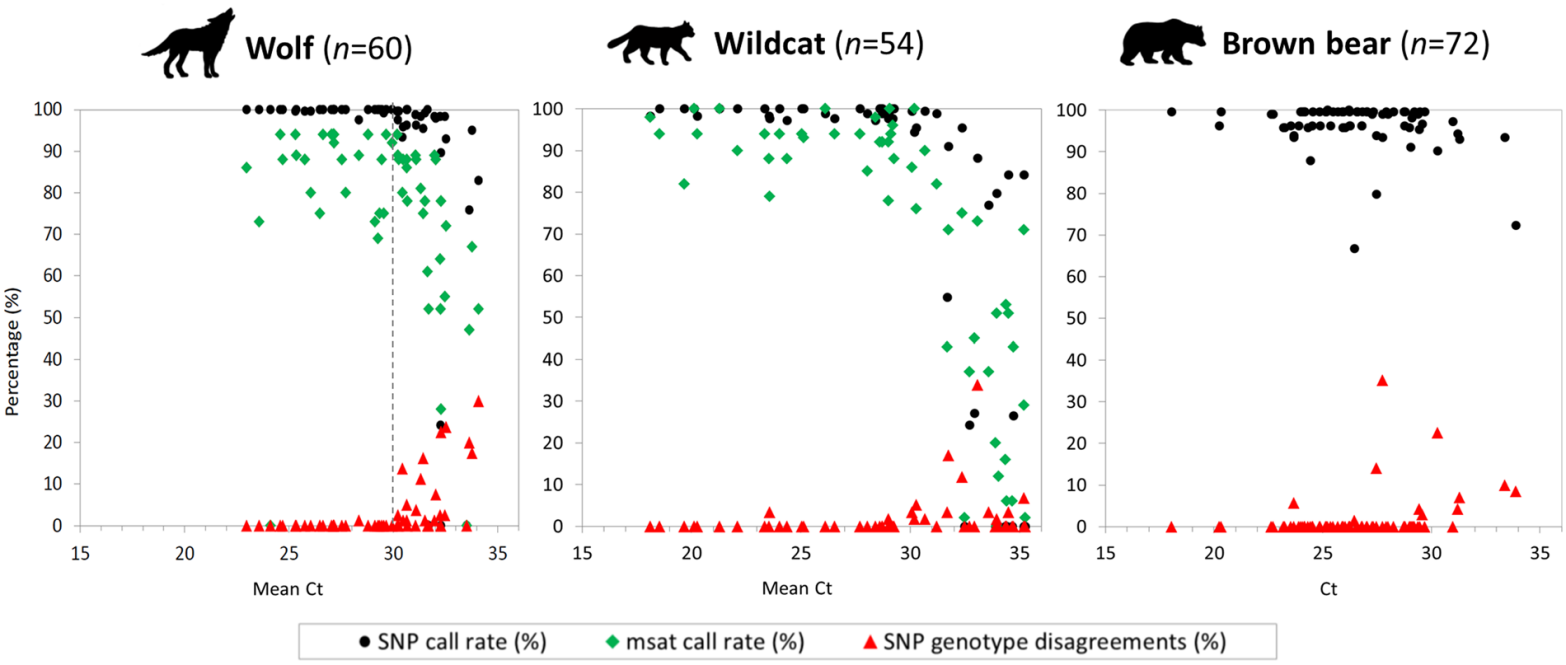
Comparison of SNP and microsatellite amplification (call rates) against Ct values measured with RT-PCR. Ct values > 30 seem to indicate a threshold after which samples show dramatically lower performance (dashed line). Microsatellite call rates for the bear samples were not available.

In order to assess sample quality and, thus, the need for replication of noninvasive samples, we compared SNP genotyping performance (SNP call rate and proportion of disagreements) and microsatellite amplification rate against cycle threshold (Ct) value from real-time PCR (RT-PCR) (Fig. 2). The percentage of disagreements across the three SNP genotype replicates increased with Ct, while the SNP and microsatellite call rates decreased with increasing Ct. Notably, samples with Ct values >30 showed dramatically lower performance.

### Performance of SNP vs. microsatellite markers

Both SNPs and microsatellites identified the same individuals, with no differences between the two marker types (Supplementary Table S3). However, we were able to generate more SNP- than microsatellite-based genotypes, namely 87% vs. 70% for the wolf and 80% vs. 54% for the cat (after applying quality thresholds of ≥70% amplified loci; Supplementary Figure S1). The bear samples showed similar success in genotype recovery for both marker types (97% vs. 99%); however, these rates are not directly comparable due to sample pre-selection and DNA preservation conditions between microsatellite and SNP genotyping analyses (see Methods).

**Figure 3:**
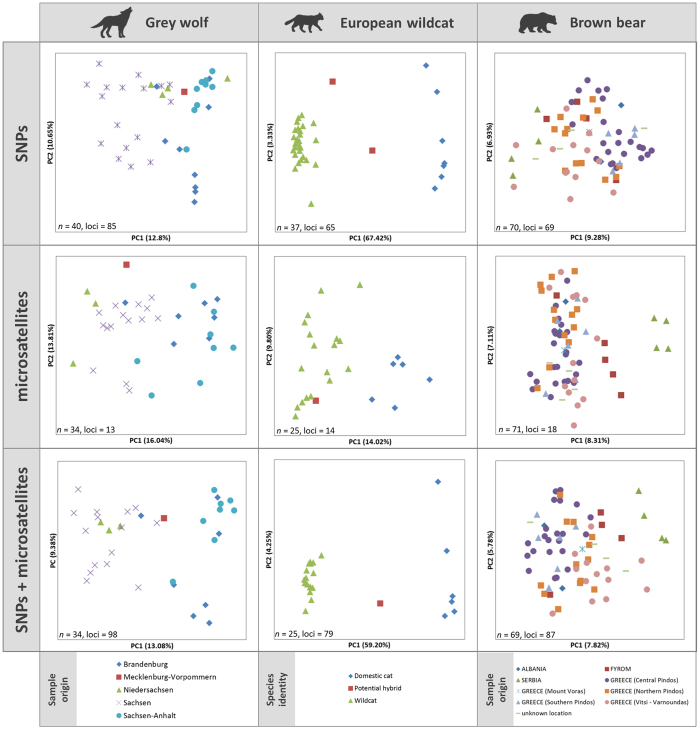
PCoA analyses of SNP and microsatellite data. Each point represents an individual’s genotype, colour-coded to its sampling region (grey wolves, brown bears) or species identification (wildcats or domestic cats, based on SNP data). In the case of the cats, two potential hybrids were identified with SNPs and one with microsatellites, due to amplification failure of one of the samples with microsatellites. Number of samples and loci included in each analysis are indicated in the lower left corner of each panel.

Assessment of genetic substructure using PCoA and STRUCTURE (Fig. 3 and Supplementary Figure S3, respectively) revealed the presence of genetic clusters that could be attributed to either sampling locations (grey wolves, brown bears) or species identity (European wildcats and domestic cats). In the PCoAs for grey wolves, individuals sampled in the same federal state in Germany appeared closer to each other than to the remaining individuals (Fig. 3, left panels), and this effect was clearer with SNP than with microsatellite data. The PCoA for wildcats and domestic cats showed two distinct clusters, one formed by wildcats and the other one by domestic cats, with SNPs being better at differentiating the two species than microsatellites (Fig. 3, middle panels). For the brown bears, the PCoA analysis of the SNP data set showed no distinct clusters, but the four Serbian samples separated from the remaining samples with the microsatellite data (Fig. 3, right panels). The STRUCTURE plots reflected results consistent with those of the PCoAs (Supplementary Figure S3); wolves and cats showed more distinct clusters with the SNP data set, while a cluster of Serbian bear samples appeared as distinct with the microsatellite data set. When combining the SNP and microsatellite data sets, differentiation for wolves and brown bears sampled in different regions increased slightly, and, in the case of the wildcat, species differentiation remained similar or improved slightly as compared to the SNP-based analyses and certainly improved as compared to the microsatellite-based analyses (Fig. 3 and Supplementary Figure S3, bottom panels).

We further tested the power to detect population structure for different numbers of SNPs and microsatellites (Supplementary Figures S6–S12). To do this, we compared the PCoAs obtained with the original SNP marker sets (85 loci for wolves, 65 loci for wildcats, and 69 loci for bears) with those obtained with decreasing numbers of SNPs (40, 20, 10, 5 loci). Subsets of markers were selected (i) randomly, (ii) based on highest heterozygosity, and (iii) highest *F*_ST_ value (only wild and domestic cats). Similarly, we compared the original microsatellite marker sets (13 for wolves, 14 for wildcats, and 18 for bears) with decreasing numbers of microsatellite loci (10, 5 loci), selected based on highest heterozygosity and highest *F*_ST_ value (the latter only for wild and domestic cats). No major differences were found among the PCoA plots obtained from randomly selected SNPs (Supplementary Figures S7, S10 and S12). While reducing the SNP panels to 40 selected loci resulted in little effect on PCoA outcomes, further reduction of loci numbers severely reduced differentiation power (Supplementary Figures S6, S8, S9 and S11).

Regarding variability estimates, the higher number of alleles per locus in microsatellites resulted in higher heterozygosity levels than those obtained for SNPs (Supplementary Table S4). For wolves and bears, heterozygosity values for different groups were almost identical for the same marker type, so no relative comparisons across markers could be made (e.g., highest or lowest heterozygosity obtained with both markers). Wildcats and domestic cats had similar heterozygosity levels based on microsatellites, but were twofold higher for domestic cats in the case of SNPs. *F*_ST_ values calculated with either marker type for each pair of groups were almost identical (wolves, *F*_ST_ = 0.10–0.05; bears, *F*_ST_ = 0.03–0.06; Supplementary Tables S5 amd S7), except in the case of the wildcats and domestic cats, as SNP markers were specifically selected to maximize differentiation between the two taxa (SNPs, *F*_ST_ = 0.79; microsatellites, *F*_ST_ = 0.13; Supplementary Table S6).

**Figure 4:**
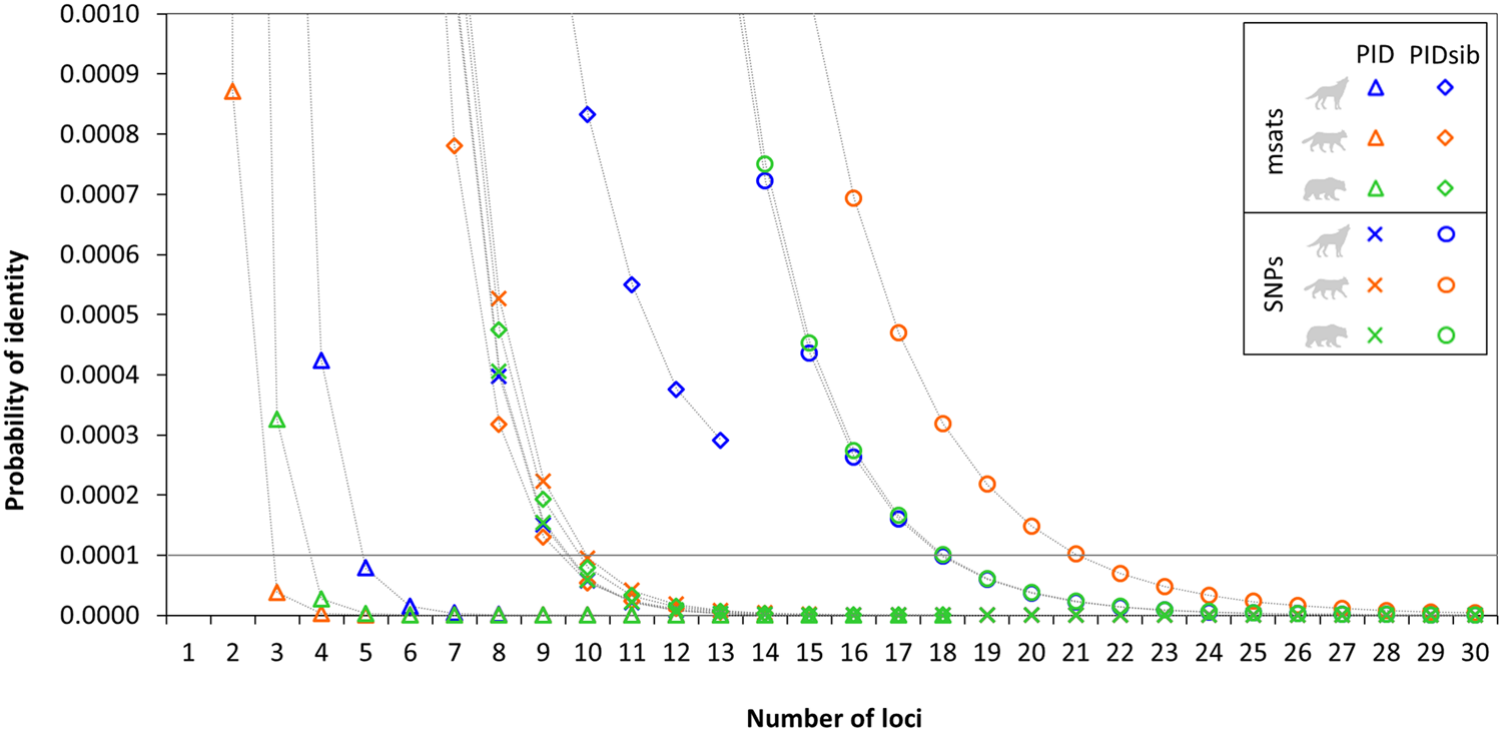
Relationship between probability of identity (PID), probability of identity between siblings (PIDsib) and the number of genotyped SNP or microsatellite loci. Loci were ranked according to highest heterozygosity (*H*_E_). A cutoff of 0.0001 was used because it is considered as sufficiently low for most applications involving natural populations^84^.

Additionally, we assessed how the probabilities of identity (PID) and the probability of identity between siblings (PIDsib) changed according to the number of loci considered (Fig. 4). In the case of the microsatellites, PID < 0.0001 is reached with three to five markers (depending on species), whereas at least 10 markers are needed to reach the same value with SNPs. The more conservative PIDsib estimates for microsatellites behaved similarly to PID values for SNPs, except in the case of the grey wolves, for which the 13 microsatellite loci used in this study were not sufficient to reach PIDsib < 0.0001. With SNP markers, PIDsib < 0.0001 was reached when 18 (wolves and bears) and 21 (wildcats) markers were employed.

## Discussion

Wildlife management decisions are increasingly informed by genetic analyses, for which noninvasive sampling has become a prominent source of DNA^11^. Some of the advantages posed by noninvasive sampling schemes include the low disturbance to the species of interest and the relative ease in which animal residues that are frequently shed or deposited into the environment can be collected^38^. However, noninvasively collected samples also present challenges due to the potentially low quantity and quality of target DNA. This problem is usually overcome by a multiple-tube approach^6, 7^, which in turn leads to a rise in analysis cost and time. In this study, we show that recently developed SNP marker panels implemented on microfluidic arrays provide reliable results for noninvasively collected samples. We further show that SNP call rates, as well as Ct values derived from RT-PCR experiments, are good indicators of sample quality and of the number of replicates that should be conducted in order to obtain reliable genotype data.

Replication of samples with very low amounts of DNA, as is often found in noninvasively collected samples, is one of the prime means to account for genotyping errors^38^. To date, the multiple-tube approach^7^ is widely applied in genetic studies based on noninvasive samples^39, 40^. Keeping the number of required replicates as low as possible is often desirable, because available funding is generally scarce and the consumption of precious DNA extracts rises with the number of replicates^41, 42^. Therefore, it was important to investigate how noninvasive samples performed when genotyped with SNP marker panels implemented using a microfluidic approach, in order to assess the reliability of the genotypes obtained and propose recommendations for its implementation. Based on our analyses, we propose a classification of the samples based on their SNP call rates, and use this as a proxy of sample quality and of the reliability of the genotype. Samples with a SNP call rate of 100% (i.e., no missing data) showed nearly no disagreements in the genotypes among the three replicates. We found only one case in which one out of the three replicates disagreed for one wolf sample at one locus, out of 74 samples that had no missing data across the three species studied. Based on this, samples that reach call rates of 100% in a first run can be excluded from further replication and the genotype considered as reliable. In the case of our study, this was the situation for 26–50% of the investigated samples (but see Discussion on sample pre-selection, below). Samples that reached 95–99% SNP call rates in first runs may need to be duplicated, since up to 17% of the generated genotypes might have disagreements. Ultimately, the researcher will need to decide which data require further validation through duplication, depending on the questions to be answered. Samples with call rates of 71–94% should be at least triplicated to detect potential errors. In our data set, we detected 34% of disagreements in this sample class. Finally, samples with call rates ≤70% may be subject to additional replication, if needed, or be discarded. When applying call rate as a proxy, it is important to consider that technical issues may very rarely hamper successful amplification. For instance, while two of the three replicates of one bear sample obtained call rates of 99%, the third replicate had a call rate of almost 0%. This is most probably explained by an air bubble introduced into the chip inlet during pipetting. This particular case led to an averaged call rate of 67% for that sample; while only one disagreement was detected between the two well-performing replicates (Fig. 1B, bears).

In this study, we included samples that had passed successful species identification tests based on mtDNA sequencing (grey wolf, wildcats) or had been successfully genotyped using microsatellite markers (brown bears), which may explain why SNP call rates were overall relatively high. Because mtDNA analysis is the primary means to determine species identity, we expect that most molecular ecology laboratories working with noninvasively collected samples will have similar pipelines of pre-selection in order to exclude samples from non-targeted species. Also, we increased DNA volume (from 1.25 µl to 3.2 µl) and STA-PCR cycles (from 14 to 18) as compared to the manufacturers’ protocol, which further aids successful amplification.

Previous studies have applied the SNP marker panels that we used here to noninvasively collected samples^35, 36, 43^. Nussberger *et al*.^36^ calculated genotyping error rates (allelic dropout and false alleles) for their SNP markers and subsequently selected hair samples adequate for SNP genotyping based on DNA concentration. Kraus *et al*.^35^ reported an overall error rate of about 1% for dilution series of tissue and blood samples (to concentrations as low as 0.2 ng/µl) and suggested that there may be no need for PCR replication. Norman *et al*.^3^ reported genotyping error rates as low as 0.00038 (based on pre-selected samples that had worked for microsatellites). A follow-up study, which investigated population size and pedigrees in bear scat samples, reported no replication for the implemented SNP genotyping approach of noninvasive faecal samples^37^. Notably, the reported error rates for SNP genotyping of noninvasive samples described so far are below microsatellite error rates using noninvasive sampling^44^.

Predicting SNP genotyping performance of noninvasive samples may be useful when selecting a subset of samples of the best quality for genotyping when many samples are available. We show here that the Ct value of RT-PCR of noninvasively collected samples may constitute a good indicator. We targeted an autosomal product of similar size to the loci being amplified in the SNP array (127 bp). Our results, based on different sample types (hair and scats), showed that both SNP and microsatellite genotyping success strongly decreased for samples with Ct values >30 and that the occurrence of SNP genotype disagreements increased. Nevertheless, it must be noted that samples with Ct values >30 occasionally performed very well. This may be due to a delay in amplification because of the presence of inhibitors or very small amounts of DNA in the extracts^45^. Also, SNP genotype disagreements of up to 1% (wolves), 3% (wildcats) or even 35% (one case in the bears) occurred for samples with Ct values <30.

Nussberger *et al*.^46^ extracted DNA from single hairs and assessed sample quality using RT-PCR quantification. They reported a minimal DNA concentration of 50 pg/µl as a threshold for SNP amplification, favouring extracts with a minimum of 200 pg/µl. Here, we decided not to attempt to determine DNA concentration because dilution series of standard DNA might not be available, particularly for non-model species, and because standards might produce different results since they are usually pristine, ultra-clean and non-degraded gDNA products. In our experience, comparing the performance of noninvasively collected samples in RT-PCR to a good quality tissue sample permitted the assessment of sample quality adequately, as was the case for the bear samples in this study.

Furthermore, we used RT-PCR results to guide the genotyping protocol conditions, in order to maximize scoring success. For samples with good quantity and quality of DNA (low Ct values) the STA pre-amplification protocol was run following the manufacturers’ protocol including 1.25 µl of DNA and 14 cycles. Samples with lower quality and quantity of DNA were performed with our modified STA protocol of 3.2 µl DNA and 18 cycles of amplification.

SNP genotyping is increasingly applied to investigate wild populations and to inform management decisions, and it is thus of utmost interest to compare the performance of novel SNP panels to more traditionally applied panels of microsatellites when genotyping noninvasively collected samples^37, 46–49^. In terms of amplification success (call rate) SNP markers performed better when compared to microsatellite markers. This increased success might be explained by the shorter size of the SNP fragments (<120 bp), which favours PCR success when using small quantities of degraded DNA templates. We found no inconsistencies in the identification of individuals based on SNP and microsatellite data, proofing the suitability of the investigated SNP panels for individual identification. We show here that approximately 20 of the most heterozygous SNPs in our data sets were sufficient to reach PIDsib < 0.0001 and thus should distinguish even closely related individuals with high certainties. Panels of approximately 12 microsatellites performed similarly well, except for the relatively homozygous Central European wolf population, where PIDsib calculations showed that inbred individuals cannot be differentiated when applying a strict PIDsib < 0.0001 cutoff.

The genotypic performance and statistical power of SNP markers as compared to standard microsatellite markers has occupied population geneticists for years (refs 50–54, for a comprehensive overview see ref. 55). Because of the bi-allelic nature of SNP markers, much higher numbers of SNP loci should be required to reach the same statistical power as with multi-allelic microsatellites^56, 57^. While many studies use thousands of SNP markers derived from high-density SNP chips or NGS data with high statistical power^14, 25, 58, 59^, others have shown that few selected SNPs may provide enough information to answer questions on individual identification, population origin or introgression^13, 24, 54, 60–62^. In summary, the number of required markers will depend on the question posed, the diagnostic power of the markers, their variability and the particular history of the population under study. Morin *et al*.^52^ propose the software POWSIM^63^ for an estimation of sample size, number of loci and types of SNPs needed for a study as well as testing the statistical power for a given SNP marker set. A pilot study that assesses the adequacy of the markers is advisable, as it is the case with microsatellites. However, to make cross-laboratory comparisons possible, the same SNP panels should be used to aid marker harmonization (see also ref. 5).

Due to marked differences in allelic richness, heterozygosity values with SNP markers were approximately half as with microsatellites. *F*_ST_ values between predefined regional subpopulations were almost identical with the two marker types, except for the wildcat, where the SNP loci were designed to maximize *F*_ST_ between wildcats and their domestic congeners. Due to the bi-allelic nature of SNP markers, a number of monomorphic loci in the SNP panels are almost inevitable, particularly when they are designed to maximize differentiation in a different population (wildcats, Switzerland^36^; bears, Scandinavia^43^) or if they are applied to a small sample size and/or a population with little genetic differentiation (wolves, compare Supplementary Table S4). Future studies with larger sample sizes and proper sampling design should further take into account differences in estimates of effective population sizes with SNP and microsatellite markers, as preliminarily tested in a simulation study^54^.

When comparing genetic structures within the sampling sets using SNPs and microsatellites, differences appeared rather moderate, except for the cat dataset. The most likely *K* values in STRUCTURE were higher for the SNP data sets for wolves and bears, indicating that the resolution power of the SNP markers might be higher in this particular case (compare Supplementary Figures S2 and S3). We tested if this was an effect of sample size, but did not find any differences when using the same individuals in both marker sets (Supplementary Figure S4). Nevertheless, these types of genetic studies are typically performed with a higher number of samples and/or with samples from more widespread locations than in the examples presented here. In some cases, the combination of both microsatellite and SNP markers may produce the best results^13, 50, 61^. When we combined the genotypes of SNP and microsatellite loci for each species, the resolution of the PCoA and STRUCTURE analyses improved slightly. This effect may be due to the increase in the number of markers, and may also be observed if the total number of SNPs is increased. Several studies have shown that raising the number of loci rather than investigating more individuals further increases the power of inferences^64, 65^.

For laboratories that process many samples in the frame of routine genetic monitoring, especially using noninvasively collected samples, SNP genotyping with microfluidic arrays may easily become the method of choice due to the reduced cost, hands-on and scoring time as compared to microsatellites. Microfluidic platforms currently enable the analysis of up to 96 SNP markers on the same number of samples in one run^66^. Considering this, the analysis of single samples may be more expensive, as single samples cannot be run alone economically. Previously, we estimated that processing 24 samples would result in similar costs either for microsatellites or SNP genotyping using the Fluidigm technology, whereas processing 240 samples would result in almost double costs and, therefore, microsatellite analysis may be the cheapest when sample sizes are small and labour costs low^5^. This is, however, not an issue in laboratories that process large numbers of samples, where a chip can readily be filled with approximately 30 samples that are triplicated, if necessary. Several studies have shown that a panel of up to 100 SNPs is sufficient to distinguish among individuals, resolve basic family structures and population origin, and even to detect fine-scale landscape relatedness^3, 46, 47, 51, 52^. If finer-scale genetic assessments are needed, additional SNP panels may easily be added to the currently available sets.

With the advent of NGS in the last decades, and the associated arrival of high-throughput SNP genotyping, analyses based on high numbers of markers are no longer an issue. A number of different genotyping platforms for diverse sample throughputs and incorporating various kinds of multiplexing capabilities and chemistries have become available^67^. Eventually, the choice of genotyping platform is largely influenced by the application and sample requirements, where cost and throughput need to be balanced individually (see also ref. 55). Here we used Fluidigms’ microfluidic array technology, as have done others seeking to SNP-genotype noninvasively collected samples^35, 46, 47^. However, additional technologies have been reported, including the MassARRAY platform (Sequenom)^49^ or SNaPshot^61^. The microfluidic arrays introduced by Fluidigm^66^ combine, in our view, several advantages. Some of the major benefits include the suitability to genotype low quality and quantity DNA, due to a pre-amplification step and the small size of the amplicons; the few microliters of DNA extract required, 3.2 µl in our adapted protocol; and the relatively straight-forward methodology, provided standard molecular laboratory setup and expertise are available. The MassARRAY technology (Sequenom) may present a comparable alternative to the microfluidic approach; however, only up to 42 SNP loci could be co-amplified with the MassARRAY platform using noninvasive samples^49^. Finally, the implementation of massive parallel sequencing has markedly improved microsatellite genotyping^68^, which may present another powerful tool for genotyping of noninvasive samples, but may also have similar constraints.

**Figure 5:**
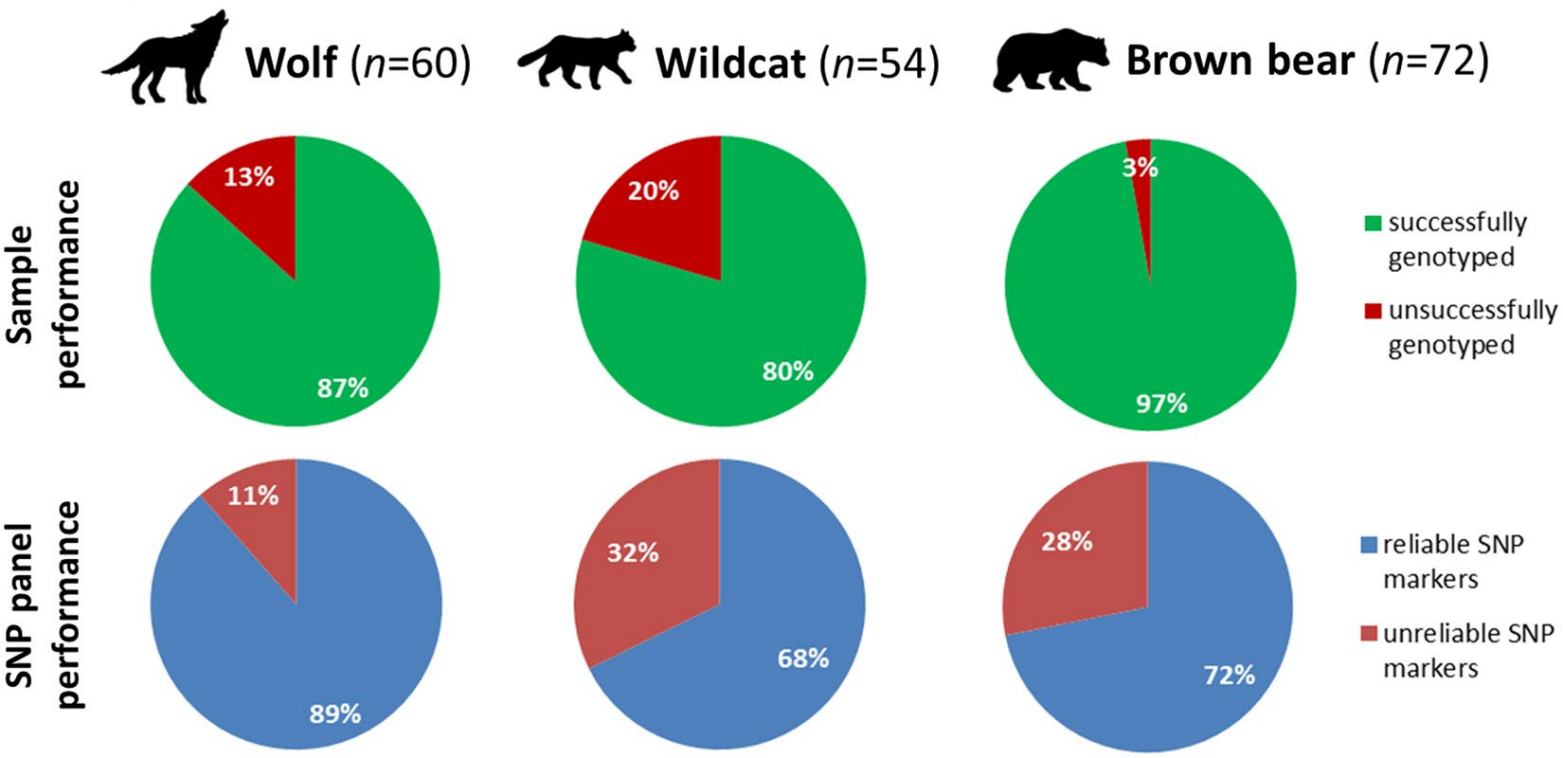
SNP genotyping performance of noninvasively collected samples. Samples were rated as ‘successfully genotyped’ if the genotype data obtained was of sufficient quality for downstream analyses by fulfilling a set of criteria (see text for details; briefly, a consensus could be derived based on a minimum of two replicates across ≥70% of loci after exclusion of badly performing loci). A SNP marker was rated as ‘reliable’ if, after genotype consensuses were obtained, ≥70% of the samples had data for that marker.

Here we have shown that noninvasively collected samples selected using proposed thresholds and treated with specific protocols may provide good quality SNP data (Fig. 5) while keeping replication to a minimum. Calculating the corresponding SNP call rate of a sample allows for the adjustment of the number of replicates that might be needed to reduce error rates or, alternatively, to discard the sample (Fig. 1). In addition, pre-evaluation of samples using RT-PCR will allow for the calibration of thresholds for the replication of samples (Fig. 2). We provide a set of recommendations for SNP genotyping using microfluidic arrays on noninvasively collected samples, as follows:

- *Costs. –* SNP genotyping may incur higher costs than microsatellite genotyping in the case of a single or a few samples, but costs dramatically decrease for larger sample sets.
- *Choice of markers. –* SNP marker selection should be preceded by a validation phase; in our experience, approximately 10% of SNP markers that performed well with tissue samples continually failed to amplify when applied to noninvasively collected samples.
- *Genotyping protocol*. – Ct values obtained from RT-PCRs can be used to determine the best SNP genotyping protocol. Generally, high quality samples with low Ct values perform well with the manufacturers’ STA pre-amplification protocol, while performance of samples with high Ct values benefited from our modified STA protocol (see Methods section).
- *Sample selection. –* The exact Ct value indicative of good SNP genotyping performance may need to be validated through a small pilot experiment. This will help to exclude low quality samples.
- *Genotyping success. –* To keep costs to a minimum, we found that the number of required replications for a sample can be determined after a first run, based on call rate (see above for thresholds). This may result in avoiding replication in the case of noninvasively collected samples of high quality, or reduce replication to the necessary minimum.
- *Reference libraries. –* We recommend setting up a reference set of high quality samples that will ideally include homozygotes and heterozygotes for all loci in the marker panel. The genotypes of these samples can be used to create a reference library, which is then applied as a reference to subsequent runs of noninvasive samples to improve clustering and facilitate scoring.

## Methods

### Sample selection and DNA extraction

We SNP-genotyped wolf scats (*n* = 60), and wildcat and domestic cat hair (*n* = 41 and *n* = 13, respectively) that were selected randomly using the *sample()* function in *R* from samples collected in the frame of the German wildlife monitoring program between 2004 and 2015 (Supplementary Table S2). These samples were mtDNA sequenced for species identification and typed with microsatellite markers (Supplementary Methods). Brown bear hair samples (*n* = 72) were analysed as part of a larger genetic study focusing on brown bears from the Balkans and were selected for SNP genotyping based on successful performance in microsatellite genotyping. Wolf scats were stored in 96% ethanol at room temperature until DNA extraction, while cat hairs were stored at room temperature between dry filter paper within a plastic sample bag containing silica as a desiccant agent until DNA extraction. We extracted DNA using the QIAamp DNA Stool Mini Kit (Qiagen) and the QIAamp DNA Investigator Kit (Qiagen), respectively. Bear hair samples were stored and extracted as described in^69^. For shipment between laboratories, bear DNA extracts were dried after microsatellite genotyping and reconstituted prior to SNP analyses.

No animals were handled or killed for this study. Noninvasive scat and hair samples were collected in compliance with the respective local and national laws.

### RT-PCR measurements

We performed RT-PCR measurements in a TOptical Gradient thermal cycler (Biometra). PCR reactions with a total volume of 10 µl contained 3.2 µl DNA, 5 µl 2x SensiFAST SYBR No-Rox Mix (Bioline), 1.4 µl molecular grade water and 0.2 µl of forward and reverse species-specific primers (10 µM each) (Table 1). Thermal cycler conditions included an initial denaturation step of 95 °C for 3 min, followed by 38 cycles of 95 °C for 5 s, 65 °C for 10 s and 72 °C for 5 s. The melting curve was performed at 60 to 95 °C, with measurements taken every 10 s with an increment of temperature (∆T) of 1 °C and a heating rate of 5 °C/s. Standards containing 10 ng/µl, 1 ng/µl, 100 pg/µl and 10 pg/µl of domestic cat and domestic dog genomic DNA (Zyagen) were included as controls. Bear samples were evaluated relative to good quality samples, as no standards were available. Samples were run alone or in duplicates, along with two no-template controls (NTC) per RT-PCR run. Results were analysed using the software qPCRsoft 3.1 (Biometra).

**Table 1:** List of primers used in this study for RT-PCR measurements.

Target species	Target region	Primer name	Sequence	Reference
European wildcat	c-Myc proto-oncogene	catcMycfor (fwd)	ACGCACAACGTCTTGGAAC	36
		catcMycrev (rev)	TGGCCTTTTTAAGGATCACC	
Grey wolf	MC1R (melanocortin 1 receptor)	MC1R.Canis.F1 (fwd)	CTGCTGGGCTCTCTCAATGG	this study
		MC1R.R1 (rev)	GCCCCAGGCTGAGGAACAG	
Brown bear	MC1R (melanocortin 1 receptor)	MC1R.CARN.F1 (fwd)	CCGGTGCCTGGAGGTGTC	this study
		MC1R.CARN.R1 (rev)	ATACATGGGCGAGTGCAGG	

### Genotyping

#### Mitochondrial DNA and microsatellite genotyping

Prior to SNP typing, the species identity of scat and hair samples was determined using mtDNA sequencing as described in previous studies (wolves^70^, wildcat^71^). Brown bear hair samples were checked macroscopically in order to avoid wild boar hairs. Unlinked autosomal microsatellite data for grey wolves and European wildcats were obtained as part of the regular genetic monitoring program implemented in our laboratory and from brown bears as part of another study^69^. Detailed protocols are provided in the Supplement and elsewhere (wolves^2^; wildcats^71^; brown bears^72^).

#### SNP genotyping

DNA samples were genotyped using 96.96 Fluidigm Dynamic Arrays (Fluidigm) with integrated fluidic circuits (IFCs). All marker panels consisted of 96 SNPs and were developed to maximize individual information (wolves^35^; brown bears^43^) or discriminate between wild and domestic taxa (wildcats^36^). The genotyping protocol involved two consecutive PCR reactions. In the first one, all 96 loci were pre-amplified in a single reaction with locus-specific primers (STA reaction) in order to generate sufficient amounts of template for the subsequent genotyping reaction. Next, fluorescently labelled allele-specific primers were used to target both alleles in a genotyping PCR. Finally, the fluorescence signal of each reaction was measured in an endpoint reader (EP1 reader, Fluidigm) in order to determine whether the individual was homozygote or heterozygote for that locus.

We slightly modified the manufacturers’ original STA protocol to facilitate the genotyping of noninvasively collected samples that typically feature low DNA quantity and quality. STA-PCRs contained 3.2 µl (instead of 1.25 µl) of DNA, 4 µl 2x QIAGEN Multiplex PCR Master Mix (Qiagen) and 0.8 µl of 10x SNPtype Assay STA primers (500 nM). Thermal conditions included an initial denaturation step of 95 °C for 15 min, followed by 18 (instead of 14 cycles) of 95 °C for 15 s and 60 °C for 4 min. STA products were diluted 1:10 (instead of 1:100) with DNA Suspension Buffer (TEKnova, PN T0221). The subsequent genotyping PCR was conducted according to the manufacturer’s protocol, but in addition to measuring the fluorescent signal after 34 cycles, a second measurement was performed after four more cycles to ensure sufficient fluorescence strength. All samples were run as triplicates along with four NTC reactions per plate to monitor for potential contamination.

#### SNP genotype scoring

We scored SNPs using the Fluidigm SNP genotyping analysis software v4.1.2, manually validating the automatically generated scatter plots. First, sample replicates with a call rate of ≤70% over all SNP loci were invalidated and thus excluded from the clustering algorithm. Next, we eliminated the loci in which at least one NTC overlapped with the samples’ genotype clusters or in which the clusters were not clearly separated from one another, as this made those loci unreliable. In addition, we invalidated NTCs that showed significant fluorescence (≥0.2) to ensure proper fluorescence data normalization over all loci. Occasional fluorescence of NTCs is a known phenomenon in the Fluidigm system, but is no cause for particular concern, because target DNA is favoured in PCRs containing sample DNA^35^. To facilitate genotype calling and improve clustering analysis, assay reference libraries were incorporated, which should typically feature good quality samples and all homozygote types.

#### Assessment of SNP genotype consistency across replicates

To prevent the influence of locus-specific performance on the assessment of replicate genotypes, we excluded loci that worked in <70% of the reactions and, in the case of the bears, also the mtDNA and Y-linked SNP loci (*n* = 4 and *n* = 2, respectively). This resulted in 16 loci, 37 and 25 loci being removed from the wolf, wildcat and brown bear data sets, respectively.

We evaluated sample performance by calculating the SNP call rate for each sample; we defined this as the percentage of called genotypes or successful amplifications over all loci for that sample. Because each sample was genotyped three times, we calculated a mean call rate for each sample as an average of call rates (Fig. 1A).

We subsequently assessed genotype consistency across replicates in relation to call rate (Fig. 1B). To do this, we counted the number of loci which agreed or disagreed on a genotype or, alternatively, had missing data across the three PCR replicates of a sample at each locus. If at least two of the PCR replicates contained missing data, the locus was counted as having insufficient/missing data. If at least one replicate disagreed, the locus was counted as having a disagreement. If all three replicates agreed on the genotype or, alternatively, if two agreed while the remaining one contained missing data, the locus was counted as having an agreement. We then calculated the percentage of loci with agreements, disagreements, or insufficient/missing data, and plotted them to the corresponding SNP call rate (per sample over all loci) (Fig. 1B). Furthermore, we investigated the variability in missing data across the three replicates in relation to the call rate. We calculated the mean of the rounded standard deviations for the percentage of missing data across all samples with the same average SNP call rate (Fig. 1C).

Given the bi-allelic nature of the loci examined with the Fluidigm technology, it is difficult to classify genotyping errors as false alleles or dropouts. Therefore, we omitted these types of error classifications in our analyses.

### Comparison of SNP vs. microsatellite performance

In order to better understand SNP performance, we compared amplification success against Ct values of RT-PCR for SNPs and microsatellites, and performed individual identification and population genetic inferences with both marker types.

#### Consensus genotypes

We determined SNP consensus genotypes for each sample over two to three replicates with a custom *R* script in the case of wolves and cats, and using ConGenR^73^ in the case of bears. The script counts how many times each allele was found over the replicates and the most common allele is assumed as true. When more than one replicate had missing data at a locus, the genotype at the respective locus was considered as missing data. Resulting consensus genotypes were checked for quality (i.e., rates of missing data) per sample and locus. In contrast to the assessment of genotype consistency across replicates described above, the quality thresholds were in this case applied to consensus data only, thus resulting in slightly differing numbers of samples and loci (Supplementary Figure S1). Samples with ≤70% SNP call rate (i.e., more than 30% missing data) were excluded from the analyses. We only examined autosomal loci here, and thus excluded mtDNA and Y-linked SNPs from the wildcat (*n* = 8 and *n* = 2, respectively) and the bear dataset (*n* = 4 and *n* = 2, respectively). This resulted in data sets comprising 52 samples and 85 loci for the grey wolf, 43 samples and 65 loci for European wildcats and domestic cats, and 70 samples and 69 loci for the brown bear. We determined the microsatellite consensus genotype for wolf and wildcat microsatellite data with the custom *R* script described above. We applied a multiple-tubes approach with four (wolf) or three (cats) PCR replicates each. Consensus genotypes with more than four missing loci, an allelic dropout rate of >0.4 and a call rate of <50% were excluded from further analyses. The microsatellite consensus genotypes for brown bears were determined as described in ref. 69. This resulted in 42 samples with data for 13 loci for the grey wolf, 29 samples and 14 loci for European wildcat and domestic cat, and 71 samples and 18 loci for the brown bear.

#### Individualization

To find matching consensus genotypes, we used the *R* package DNA TOOLS^74^ in the case of wolves and cats and ConGenR^73^ in the case of bears. We accepted one mismatch at one locus to consider genotypes as belonging to the same individual. Based on SNP data, we identified 41 individual grey wolves, 39 individual wildcats and domestic cats and 70 individual brown bears. Using microsatellite data, we identified 34 individual grey wolves, 25 individual wildcats and domestic cats and 71 individual brown bears (see Supplementary Figure S1 for details). The difference in the number of individuals identified using SNP- or microsatellite-based genotypes was due to microsatellite amplification failure. We found that all successfully genotyped samples represented a different individual, except for seven grey wolf individuals which were each represented by multiple (up to four) samples, and four wildcat individuals which were each represented by two samples (Supplementary Table S3). These results were the same with SNPs or microsatellites, except for three wolf samples that did not amplify with microsatellites. Among wolves and cats, we found four pairs of genotypes differentiated by single mismatches in the SNP data sets, and four pairs of genotypes differentiated by single mismatches in the microsatellite data sets, which were considered to belong to the same individual. No matching genotypes were found in the brown bear data sets, consistent with the fact that all samples had been previously individualized using a panel of 18 microsatellites in the course of a previous study. All matching genotypes were checked against metadata, like sampling dates and locations, and no contradictory evidence was found.

#### PCoA

We used the PCoA implemented in GenAlEx v.6.5^75, 76^ in order to infer genetic structure in our genotype datasets. We identified one outlier in the wolf and two in the cat data sets that, on closer inspection, had low SNP call rates (between 80–71%) and were therefore excluded from the figures and further analysis (Supplementary Figure S5 with outliers). No outliers were identified using the microsatellite data sets. To ascertain relative statistical power of SNP markers, we performed PCoA analyses for sets of decreasing number of SNPs (40, 20, 10, 5 loci) selected (i) randomly (3 times each case); (ii) based on highest heterozygosity; and (iii) highest *F*_ST_ value (only wild and domestic cats). Furthermore, we tested subsets of microsatellite markers (10, 5 loci) selected based on highest heterozygosity and highest *F*_ST_ (the latter only for wild and domestic cats). We assumed that, in studies in which a minimum number of markers are desired and a pool of available markers to select from exists, the markers chosen may either be the most heterozygous ones or, in the case of hybridization studies, those that best allow to differentiate two taxa.

#### Bayesian clustering with STRUCTURE

We tested for population structure using the Bayesian clustering algorithm implemented in STRUCTURE^77^. After an initial burn-in of 250,000 steps, 500,000 MCMCs were run using the admixture model with correlated allele frequencies with no prior information. Ten independent iterations were run for each *K* = 1–10 and combined using the GREEDY or LARGEKGREEDY algorithm as implemented in CLUMPP^78^. The Evanno method^79^ as implemented in STRUCTURE HARVESTER^80^ was used to select the most likely *K* value (Supplementary Figure S2).

#### Basic population genetic parameters and probabilities of identity

Analyses of basic population genetic measures and calculations of probabilities of identity were performed with GenAlEx v6.5^76^. We calculated the mean number of different alleles (*Na*), observed heterozygosity (*H*_O_) and (unbiased) expected heterozygosity (*H*_E_) for sampling groups with *n* ≥ 5 and for total sample pools of each species. Pairwise *F*_ST_ calculations were performed using AMOVA for sampling groups with *n* ≥ 5 and significance assessed using a permutation approach^81^. We calculated probability of identity (PID) and probability of identity between siblings (PIDsib) for SNP and microsatellite loci for all three investigated species according to^82–84^. For PID and PIDsib calculations loci were ranked according to highest (unbiased) expected heterozygosity (*H*_E_).

#### Data availability

The datasets generated and analysed during the current study are available from the corresponding authors on reasonable request.

## Electronic supplementary material

Supplementary Information

## References

[1] Valière N (2003). Long-distance wolf recolonization of France and Switzerland inferred from non-invasive genetic sampling over a period of 10 years. Anim. Conserv..

[2] Harms V, Nowak C, Carl S, Muñoz-Fuentes V (2015). Experimental evaluation of genetic predator identification from saliva traces on wildlife kills. J. Mammal..

[3] Norman AJ, Spong G (2015). Single nucleotide polymorphism-based dispersal estimates using noninvasive sampling. Ecol. Evol..

[4] Steyer K (2016). Large-scale genetic census of an elusive carnivore, the European wildcat (*Felis s. silvestris*). Conserv. Genet..

[5] deGroot GA (2016). Decades of population genetic research reveal the need for harmonization of molecular markers. The grey wolf *Canis lupus* as a case study. Mamm. Rev..

[6] Navidi W, Arnheim N, Waterman MS (1992). A multiple-tubes approach for accurate genotyping of very small DNA samples by using PCR: statistical considerations. Am. J. Hum. Genet..

[7] Taberlet P, Waits LP, Luikart G (1999). Noninvasive genetic sampling: look before you leap. Trends Ecol. Evol. (Amst.).

[8] Broquet T, Petit E (2004). Quantifying genotyping errors in noninvasive population genetics. Mol. Ecol..

[9] Gagneux P, Boesch C, Woodruff DS (1997). Microsatellite scoring errors associated with noninvasive genotyping based on nuclear DNA amplified from shed hair. Mol. Ecol..

[10] Goossens B, Waits LP, Taberlet P (1998). Plucked hair samples as a source of DNA: reliability of dinucleotide microsatellite genotyping. Mol. Ecol..

[11] Smith O, Wang J (2014). When can noninvasive samples provide sufficient information in conservation genetics studies?. Mol. Ecol. Resour..

[12] Brumfield RT, Beerli P, Nickerson DA, Edwards SV (2003). The utility of single nucleotide polymorphisms in inferences of population history. Trends Ecol. Evol. (Amst.).

[13] Muñoz-Fuentes V, Vilà C, Green AJ, Negro JJ, Sorenson MD (2007). Hybridization between white-headed ducks and introduced ruddy ducks in Spain. Mol. Ecol..

[14] Willing E-M (2010). Genome-wide single nucleotide polymorphisms reveal population history and adaptive divergence in wild guppies. Mol. Ecol..

[15] vonHoldt BM (2011). A genome-wide perspective on the evolutionary history of enigmatic wolf-like canids. Genome Res..

[16] Rutledge LY, Wilson PJ, Klütsch CF, Patterson BR, White BN (2012). Conservation genomics in perspective. A holistic approach to understanding *Canis* evolution in North America. Biol. Cons..

[17] Natarajan C (2015). Convergent evolution of hemoglobin function in high-altitude Andean waterfowl involves limited parallelism at the molecular sequence level. PLoS Genet..

[18] Grover A, Sharma PC (2016). Development and use of molecular markers: past and present. Crit. Rev. Biotechnol..

[19] Morin PA, Luikart G, Wayne RK (2004). & the SNP workshop group. SNPs in ecology, evolution and conservation. Trends Ecol. Evol. (Amst.).

[20] Winton CL (2013). Genetic diversity and phylogenetic analysis of native mountain ponies of Britain and Ireland reveals a novel rare population. Ecol. Evol..

[21] Bradbury IR (2015). Transatlantic secondary contact in Atlantic Salmon, comparing microsatellites, a single nucleotide polymorphism array and restriction-site associated DNA sequencing for the resolution of complex spatial structure. Mol. Ecol..

[22] Schlotterer C (2004). The evolution of molecular markers - just a matter of fashion?. Nat. Rev. Genet..

[23] Jonker RM (2012). The development of a genome wide SNP set for the Barnacle goose *Branta leucopsis*. PLoS ONE.

[24] Muñoz I (2015). Reduced SNP panels for genetic identification and introgression analysis in the dark honey bee (*Apis mellifera mellifera*). PLoS ONE.

[25] Pellegrino I (2016). Development of SNP markers for population structure and phylogeography characterization in little owl (*Athene noctua*) using a genotyping-by-sequencing approach. Conserv. Genet. Resour..

[26] Garvin MR, Saitoh K, Gharrett AJ (2010). Application of single nucleotide polymorphisms to non-model species: a technical review. Mol. Ecol. Resour..

[27] Helyar SJ (2011). Application of SNPs for population genetics of nonmodel organisms: new opportunities and challenges. Mol. Ecol. Resour..

[28] Trucchi E (2016). BsRADseq: screening DNA methylation in natural populations of non-model species. Mol. Ecol..

[29] Albrechtsen A, Nielsen FC, Nielsen R (2010). Ascertainment biases in SNP chips affect measures of population divergence. Mol. Biol. Evol..

[30] Brandstrom M, Ellegren H (2008). Genome-wide analysis of microsatellite polymorphism in chicken circumventing the ascertainment bias. Genome Res..

[31] Vali U, Einarsson A, Waits LP, Ellegren H (2008). To what extent do microsatellite markers reflect genome-wide genetic diversity in natural populations?. Mol. Ecol..

[32] Queiros J (2015). Effect of microsatellite selection on individual and population genetic inferences: an empirical study using cross-specific and species-specific amplifications. Mol. Ecol. Resour..

[33] Morin PA, McCarthy M (2007). Highly accurate SNP genotyping from historical and low-quality samples. Mol. Ecol. Notes.

[34] Ogden R (2011). Unlocking the potential of genomic technologies for wildlife forensics. Mol. Ecol. Resour..

[35] Kraus RHS (2015). A single-nucleotide polymorphism-based approach for rapid and cost-effective genetic wolf monitoring in Europe based on noninvasively collected samples. Mol. Ecol. Resour..

[36] Nussberger B, Wandeler P, Camenisch G (2014). A SNP chip to detect introgression in wildcats allows accurate genotyping of single hairs. Eur. J. Wildl. Res..

[37] Spitzer R, Norman AJ, Schneider M, Spong G (2016). Estimating population size using single-nucleotide polymorphism-based pedigree data. Ecol. Evol..

[38] Waits LP, Paetkau D (2005). Noninvasive genetic sampling tools for wildlife biologists. A review of applications and recommendations for accurate data collection. J. Wildlife Manage..

[39] Pompanon F, Bonin A, Bellemain E, Taberlet P (2005). Genotyping errors: causes, consequences and solutions. Nat. Rev. Genet..

[40] Clozato CL, Moraes-Barros Nd, Santos FR, Morgante JS (2014). Historical and non-invasive samples. A study case of genotyping errors in newly isolated microsatellites for the lesser anteater (*Tamandua tetradactyla* L., Pilosa). Mol. Ecol. Resour..

[41] Paetkau D (2003). An empirical exploration of data quality in DNA-based population inventories. Mol. Ecol..

[42] Frantz AC (2003). Reliable microsatellite genotyping of the Eurasian badger (*Meles meles*) using faecal DNA. Mol. Ecol..

[43] Norman AJ, Street NR, Spong G (2013). De novo SNP discovery in the Scandinavian brown bear (*Ursus arctos*). PLoS ONE.

[44] Valière N (2007). Importance of a pilot study for non-invasive genetic sampling: genotyping errors and population size estimation in red deer. Conserv. Genet..

[45] King C, Debruyne R, Kuch M, Schwarz C, Poinar H (2009). A quantitative approach to detect and overcome PCR inhibition in ancient DNA extracts. BioTechniques.

[46] Nussberger B, Wandeler P, Weber D, Keller LF (2014). Monitoring introgression in European wildcats in the Swiss Jura. Conserv. Genet..

[47] Norman AJ (2017). Landscape relatedness. Detecting contemporary fine-scale spatial structure in wild populations. Landscape Ecol.

[48] Cronin MA (2014). Molecular phylogeny and SNP variation of polar bears (*Ursus maritimus*), brown bears (*U. arctos*), and black bears (*U. americanus*) derived from genome sequences. J. Hered..

[49] Goossens B (2016). Habitat fragmentation and genetic diversity in natural populations of the Bornean elephant. Implications for conservation. Biol. Cons..

[50] Puckett EE, Eggert LS (2016). Comparison of SNP and microsatellite genotyping panels for spatial assignment of individuals to natal range. A case study using the American black bear (*Ursus americanus*). Biol. Cons..

[51] Tokarska M (2009). Effectiveness of microsatellite and SNP markers for parentage and identity analysis in species with low genetic diversity: the case of European bison. Heredity.

[52] Morin PA, Martien KK, Taylor BL (2009). Assessing statistical power of SNPs for population structure and conservation studies. Mol. Ecol. Resour..

[53] Smith CT (2011). Impacts of marker class bias relative to locus-specific variability on population inferences in Chinook Salmon. A comparison of single-nucleotide polymorphisms with short tandem repeats and allozymes. Trans. Am. Fish. Soc..

[54] Antao T, Pérez-Figueroa A, Luikart G (2011). Early detection of population declines: high power of genetic monitoring using effective population size estimators. Evol. Appl..

[55] Puckett EE (2017). Variability in total project and per sample genotyping costs under varying study designs including with microsatellites or SNPs to answer conservation genetic questions. Conserv. Genet. Resour..

[56] Hoban S (2014). Comparative evaluation of potential indicators and temporal sampling protocols for monitoring genetic erosion. Evol. Appl..

[57] Schopen GC, Bovenhuis H, Visker M, van Arendonk JA (2008). Comparison of information content for microsatellites and SNPs in poultry and cattle. Anim. Genet..

[58] Saura M (2013). Genome-wide estimates of coancestry and inbreeding in a closed herd of ancient Iberian pigs. PLoS ONE.

[59] Pilot M (2014). Genome-wide signatures of population bottlenecks and diversifying selection in European wolves. Heredity.

[60] Seeb LW (2011). Single nucleotide polymorphisms across a species’ range: implications for conservation studies of Pacific salmon. Mol. Ecol. Resour..

[61] Fabbri E (2012). Comparison of single nucleotide polymorphisms and microsatellites in non-invasive genetic monitoring of a wolf population. Arch. Biol. Sci..

[62] Seddon JM, Parker HG, Ostrander EA, Ellegren H (2005). SNPs in ecological and conservation studies: a test in the Scandinavian wolf population. Mol. Ecol..

[63] Ryman N, Palm S (2006). POWSIM. A computer program for assessing statistical power when testing for genetic differentiation. Mol. Ecol. Notes.

[64] Landguth EL (2012). Effects of sample size, number of markers, and allelic richness on the detection of spatial genetic pattern. Mol. Ecol. Resour..

[65] Hoban SM, Gaggiotti OE, Bertorelle G (2013). The number of markers and samples needed for detecting bottlenecks under realistic scenarios, with and without recovery. A simulation‐based study. Mol. Ecol..

[66] Wang J (2009). High-throughput single nucleotide polymorphism genotyping using nanofluidic Dynamic Arrays. BMC Genomics.

[67] Perkel J (2008). SNP genotyping. Six technologies that keyed a revolution. Nat. Methods.

[68] Barba Mde (2017). High-throughput microsatellite genotyping in ecology: improved accuracy, efficiency, standardization, and success with low-quantity and degraded DNA. Mol. Ecol. Resour..

[69] Karamanlidis AA, Hernando MdG, Krambokoukis L, Gimenez O (2015). Evidence of a large carnivore population recovery. Counting bears in Greece. J. Nat. Conserv..

[70] Lesniak I (2017). Population expansion and individual age affect endoparasite richness and diversity in a recolonising large carnivore population. Sci. Rep..

[71] Steyer K (2013). Hair trapping with valerian-treated lure sticks as a tool for genetic wildcat monitoring in low-density habitats. Eur. J. Wildl. Res..

[72] Karamanlidis AA (2012). Genetic diversity, structure, and size of an endangered brown bear population threatened by highway construction in the Pindos Mountains, Greece. Eur. J. Wildl. Res..

[73] Lonsinger R, Waits LP (2015). ConGenR: rapid determination of consensus genotypes and estimates of genotyping errors from replicated genetic samples. Conserv. Genet. Resour..

[74] Tvedebrink T, Eriksen PS, Curran JM, Mogensen HS, Morling N (2012). Analysis of matches and partial-matches in a Danish STR data set. Forensic. Sci. Int. Genet..

[75] Peakall R, Smouse PE (2006). GENALEX 6: genetic analysis in Excel. Population genetic software for teaching and research. Mol. Ecol. Notes.

[76] Peakall R, Smouse PE (2012). GenAlEx 6.5: genetic analysis in Excel. Population genetic software for teaching and research - an update. Bioinformatics.

[77] Pritchard JK, Stephens M, Donnelly P (2000). Inference of population structure using multilocus genotype data. Genetics.

[78] Jakobsson M, Rosenberg NA (2007). CLUMPP: a cluster matching and permutation program for dealing with label switching and multimodality in analysis of population structure. Bioinformatics.

[79] Evanno G, Regnaut S, Goudet J (2005). Detecting the number of clusters of individuals using the software STRUCTURE: a simulation study. Mol. Ecol..

[80] Earl DA, vonHoldt BM (2012). STRUCTURE HARVESTER: a website and program for visualizing STRUCTURE output and implementing the Evanno method. Conserv. Genet. Resour..

[81] Excoffier L, Smouse PE, Quattro JM (1992). Analysis of molecular variance inferred from metric distances among DNA haplotypes. Application to human mitochondrial DNA restriction data. Genetics.

[82] Taberlet P, Luikart G (1999). Non-invasive genetic sampling and individual identification. Biol. J. Linn. Soc..

[83] Peakall R, Ebert D, Cunningham R, Lindenmayer D (2006). Mark-recapture by genetic tagging reveals restricted movements by bush rats (*Rattus fuscipes*) in a fragmented landscape. J. Zool. (Lond.).

[84] Waits LP, Luikart G, Taberlet P (2001). Estimating the probability of identity among genotypes in natural populations: cautions and guidelines. Mol. Ecol..

