# Supplementary material for: Assessing SNP genotyping of noninvasively collected wildlife samples using microfluidic arrays
Source: Sci Rep. 2017 Sep 7;7:10768. doi: 10.1038/s41598-017-10647-w (PMC5589735; doi:10.1038/s41598-017-10647-w)
Supplement: Supplementary file 1 — Supplementary Information [file 41598_2017_10647_MOESM1_ESM.pdf]

# Supplementary Information

## Assessing SNP genotyping of noninvasively collected wildlife samples using microfluidic arrays

Alina von Thaden, Berardino Cocchiararo, Anne Jarausch, Hannah Jüngling, Alexandros A. Karamanlidis, Annika Tiesmeyer, Carsten Nowak, Violeta Muñoz-Fuentes

### Index

**Supplementary Methods** Protocols used for mitochondrial DNA sequencing and microsatellite genotyping.

**Supplementary Table S1** List of mtDNA primers used in this study for species identification.

**Supplementary Table S2** Samples used in this study. For some samples the sampling location (country and/or origin) is unknown (-). M, male; F, female; U, unknown sex.

**Supplementary Figure S1** Overview of numbers of samples and loci included in each analysis based on quality criteria.

**Supplementary Table S3** Overview of individuals that were represented by multiple samples in SNP and microsatellite data sets. One mismatch at one locus was accepted to consider two genotypes as belonging to the same individual. Note that brown bear samples had been individualized using microsatellites in the course of a previous study and consequently no matching genotypes were found in this study. *n*, number of samples; f.a., sample failed in microsatellite amplification; NA, matching not available due to failed microsatellite amplification of one of the samples.

**Supplementary Table S4** Genetic variability in grey wolves, European wildcats and brown bears for SNPs and microsatellites. *n*, sample size; *Na*, mean number of different alleles over loci; $H_O$, observed heterozygosity; $H_E$; expected heterozygosity; ML, number of monomorphic loci. For sample groups with $n \leq 5$ no calculations were performed (5 grey wolves, 2 potential wildcat hybrids, 16 brown bears).

**Supplementary Table S5** Pairwise $F_{ST}$ values for grey wolves with microsatellite data (above the diagonal, $n$ = 30 samples and 13 loci) and SNP data (below the diagonal, $n$ = 35 samples and 85 loci). All samples were collected in Germany. Only groups with $n > 5$ were considered (5 wolves from 2 locations excluded). Probability values were based on 999 permutations; $*p \leq 0.05$; $**p \leq 0.01$; $***p \leq 0.001$.

**Supplementary Table S6** Pairwise $F_{ST}$ values for European wildcats and domestic cats with microsatellite data (above the diagonal, *n* = 24 samples and 14 loci) and SNP data (below the diagonal, *n* = 35 samples and 65 loci). Note that the SNP panel we used here was designed to detect hybridization of wildcats and domestic cats (that is, maximize differentiation). All samples were collected in Germany. Potential hybrids (based on SNP data) were excluded from these analyses (*n* = 1 for msats, *n* = 2 for SNPs). Probability values were based on 999 permutations; $***p \leq 0.001$.

**Supplementary Table S7** Pairwise $F_{ST}$ values for brown bears with microsatellite data (above the diagonal, *n* = 55 samples and 18 loci) and SNP data (below the diagonal, *n* = 55 samples and 69 loci). All samples were collected in Greece. Only groups with $n > 5$ were considered (16 bears from 5 locations excluded). Probability values were based on 999 permutations; n.s., not significant; $*p \leq 0.05$; $**p \leq 0.01$; $***p \leq 0.001$. Negative values were converted to zero.

**Supplementary Figure S2** Delta *K* for STRUCTURE runs corresponding to Supplementary Figure S3.

**Supplementary Figure S3** STRUCTURE plots showing results for the most likely *K* (***), second most likely *K* (**) and third most likely *K* (*) as calculated with the Evanno method based on SNP and microsatellite genotypes and their combination. Colour-coded bars below the STRUCTURE plots correspond to the sample groupings based on sampling region (grey wolves, brown bears) or species identification (wildcats or domestic cats, based on SNP data).

**Supplementary Figure S4** STRUCTURE plots showing results for the most likely *K* (***), second most likely *K* (**) and third most likely *K* (*) (upper panels) as calculated with the Evanno method (lower panels, respectively) based on SNP and microsatellite data sets. Colour-coded bars below the STRUCTURE plots correspond to the sample groupings based on sampling region (grey wolves, brown bears) or species identification (wildcats and domestic cats, based on SNP data).

**Supplementary Figure S5** PCoA for wolves and wildcats showing outliers (SNP data, original data set). Further examination of these samples indicated low SNP call rates (wolf 71%; wildcats 77%, 78%). These samples were removed from the figure in the main text, Figure 3, and from further analyses.

**Supplementary Figure S6** PCoA analyses for subsets of SNP and microsatellite markers used in this study to genotype grey wolves. Each point represents an individual's genotype, colour-coded to its sampling region. Subsets of loci were selected based on highest heterozygosity ($H_E$) for each locus.

**Supplementary Figure S7** PCoA analyses for subsets of SNP markers used in this study to genotype grey wolves. Each point represents an individual's genotype, colour-coded to its sampling region. Subsets of loci were selected randomly; three times each case (a, b, c).

**Supplementary Figure S8** PCoA analyses for subsets of SNP and microsatellite markers used in this study to genotype European wildcats, domestic cats and hybrids. Each point represents an individual's genotype, colour-coded to species identity. Subsets of loci were selected based on highest heterozygosity ($H_E$) for each locus.

**Supplementary Figure S9** PCoA analyses for subsets of SNP and microsatellite markers used in this study to genotype European wildcats, domestic cats and hybrids. Each point represents an individual's genotype, colour-coded to species identity. Subsets of loci were selected based on highest $F_{ST}$ for each locus.

**Supplementary Figure S10** PCoA analyses for subsets of SNP markers used in this study to genotype European wildcats, domestic cats and hybrids. Each point represents an individual's genotype, colour-coded to species identity. Subsets of loci were selected randomly; three times each case (a, b, c).

**Supplementary Figure S11** PCoA analyses for subsets of SNP and microsatellite markers used in this study to genotype brown bears. Each point represents an individual's genotype, colour-coded to its sampling region. Subsets of loci were selected based on highest heterozygosity ($H_E$) for each locus.

**Supplementary Figure S12** PCoA analyses for subsets of SNP markers used in this study to genotype brown bears. Each point represents an individual's genotype, colour-coded to its sampling region. Subsets of loci were selected randomly; three times each case (a, b, c).

## Supplementary Methods

### *Mitochondrial DNA sequencing*

Brown bear hair samples were checked macroscopically for species identification, in order to avoid wild boar hairs. Grey wolf scats and cat hairs were checked for species identity using mtDNA sequencing in order to avoid samples from other species (mainly, fox, dog or domestic cat). PCR reactions of 15 µl contained 3 µl DNA, 1.5 µl 10x Standard Taq Buffer (New England BioLabs), 1.8 µl of 25 mM $MgCl_2$, 0.2 µl of BSA (10 µg/µl), 1.2 µl of 2.5 mM dNTPs, 0.5 µl of species specific forward and reverse primers (10 µM) (Supplementary Table S1), 0.2 µl of Taq DNA polymerase (5 U/µl) (New England BioLabs) and 6.1 µl of molecular grade water. PCRs were performed in a T1 plus Thermocycler (Biometra). Initial denaturation was at 95 °C for 3 min, followed by 35 cycles of 94 °C for 30 s, 54 °C for 30 s, and 72 °C for 1 min and a final extension at 72 °C for 10 min. PCR products were purified with 2 µl Exonuclease I and FastAP™ Thermosensitive Alkaline Phosphatase mixture (1:2; Thermo Scientific) at 37 °C for 15 min, followed by 80 °C for 15 min and diluted 1:20 (scats) or 1:40 (hairs). Sequencing was performed using the BigDye Terminator 3.1 Cycle Sequencing Kit (Applied Biosciences) using a cycling protocol which involved an initial denaturation step at 95 °C for 60 s, followed by 30 cycles of 10 s at 96 °C, 10 s at 50 °C and 2 min at 60 °C. The products were purified using ABI-XTerminator beads (Applied Biosystems) and separated on an ABI 3730 DNA Analyzer (Applied Biosystems). Sequences of wolves and wildcats were aligned with Geneious v7.1.8[1] and aligned to our laboratory reference samples to identify haplotypes.

**Supplementary Table S1** List of mtDNA primers used in this study for species identification.

| Target species | Target region | Primer Name | Sequence | Reference |
|---|---|---|---|---|
| European wildcat | Control region | LF4 (fwd) | GACATAATAGTGCTTAATCGTGC | [2] |
| | | H16498 (rev) | CCTGAAGTAAGAACCAGATG | [3] |
| Grey wolf | Control region | L15995 (fwd) | CTCCACTATCAGCACCCAAAG | [4] |
| | | H16498 (rev) | CCTGAAGTAAGAACCAGATG | [3] |

*Microsatellite genotyping*

Unlinked autosomal microsatellite data for grey wolves and European wildcats were obtained as part of the regular genetic monitoring conducted in our laboratory. Brown bear microsatellite genotyping data was obtained from collaborators in Greece[5]. The markers and laboratory procedures are described elsewhere (wolves, [6]; wildcats, [7]; brown bears, [8]). Briefly, a multiple-tubes approach was applied for wolves and wildcats, as is common practice for noninvasive samples, including three (wildcat, domestic cat) or four (grey wolf) replicates per sample. Each microsatellite PCR of 10 µl consisted of 2X HotStarTaq Master Mix (Qiagen), 0.2 µM of each primer, 2 ng BSA and 3.7-3.8 µl of DNA. PCRs were performed in a T1 plus Thermocycler (Biometra). Initial denaturation was set to 95 °C for 3 min, followed by 4-5 cycles of 94 °C for 30 s, 60 °C for 90 s, and 72 °C for 60 s; 5 cycles of 94 °C for 30 s, 58 °C for 90 s, and 72 °C for 60 s; 5 cycles of 94 °C for 30 s, 54 °C for 90 s, and 72 °C for 60 s; and 20-25 cycles of 94 °C for 30 s, 50 °C for 90 s, and 72 °C for 60 s. Final extension was at 72 °C for 30 min. PCR products were diluted 1:5 prior to sequencing. Fragment length analysis was performed on an ABI 3730 DNA Analyzer (Life Technologies) and fragment sizes were determined using the software GeneMarker v1.90 (Softgenetics LLC) by comparison to internal LIZ size standards. The details of brown bear microsatellite genotyping are described in [5]. The microsatellite call rate for each sample was calculated as the percentage of successfully amplified PCR reactions over all replicates of a sample and over all investigated loci.

**Supplementary Table S2** Samples used in this study. For some samples the sampling location (country and/or origin) is unknown (-). M, male; F, female; U, unknown sex.

| Sample ID | Species | Sample Type | Collection Date | Country | Federal State/Region | Sex |
|---|---|---|---|---|---|---|
| WE121 | *C. lupus* | scat | 20130514 | Germany | Sachsen | M |
| WE122 | *C. lupus* | scat | 20130831 | Germany | Sachsen | U |
| WE124 | *C. lupus* | scat | 20130527 | Germany | Sachsen | U |
| WE125 | *C. lupus* | scat | 20131213 | Germany | Brandenburg | F |
| WE126 | *C. lupus* | scat | 20131213 | Germany | Brandenburg | M |
| WE136 | *C. lupus* | scat | 20040106 | Germany | Sachsen | F |
| WE137 | *C. lupus* | scat | 20040112 | Germany | Sachsen | U |
| WE138 | *C. lupus* | scat | 20040113 | Germany | Sachsen | U |
| WE139 | *C. lupus* | scat | 20040117 | Germany | Sachsen | U |
| WE141 | *C. lupus* | scat | 20120610 | Germany | Brandenburg | F |
| WE142 | *C. lupus* | scat | 20120610 | Germany | Brandenburg | M |
| WE160 | *C. lupus* | scat | 20140319 | Germany | Sachsen-Anhalt | U |
| WE161 | *C. lupus* | scat | 20140228 | Germany | Sachsen-Anhalt | U |
| WE162 | *C. lupus* | scat | 20131212 | Germany | Sachsen-Anhalt | M |
| WE163 | *C. lupus* | scat | 20140205 | Germany | Sachsen-Anhalt | F |
| WE164 | *C. lupus* | scat | 20140124 | Germany | Sachsen-Anhalt | F |
| WE165 | *C. lupus* | scat | 20140208 | Germany | Sachsen-Anhalt | U |
| WE166 | *C. lupus* | scat | 20140130 | Germany | Sachsen-Anhalt | F |
| WE167 | *C. lupus* | scat | 20140228 | Germany | Sachsen-Anhalt | F |
| WE168 | *C. lupus* | scat | 20140204 | Germany | Sachsen-Anhalt | U |
| WE169 | *C. lupus* | scat | 20140129 | Germany | Sachsen-Anhalt | M |
| WE170 | *C. lupus* | scat | 20140319 | Germany | Sachsen-Anhalt | M |
| WE171 | *C. lupus* | scat | 20140307 | Germany | Sachsen-Anhalt | M |
| WE173 | *C. lupus* | scat | 20140129 | Germany | Sachsen-Anhalt | M |
| WE174 | *C. lupus* | scat | 20140107 | Germany | Sachsen-Anhalt | F |
| WE175 | *C. lupus* | scat | 20140117 | Germany | Sachsen-Anhalt | M |
| WE191 | *C. lupus* | scat | 20140304 | Germany | Niedersachsen | U |
| WE192 | *C. lupus* | scat | 20130826 | Germany | Niedersachsen | U |
| WE196 | *C. lupus* | scat | 20130415 | Germany | Niedersachsen | U |
| WE197 | *C. lupus* | scat | 20130325 | Germany | Niedersachsen | M |
| WE199 | *C. lupus* | scat | 20140428 | Germany | Mecklenburg-Vorpommern | M |
| WE200 | *C. lupus* | scat | 20130427 | Germany | Niedersachsen | F |
| WE201 | *C. lupus* | scat | 20140122 | Germany | Brandenburg | M |
| WE202 | *C. lupus* | scat | 20140210 | Germany | Brandenburg | M |
| WE203 | *C. lupus* | scat | 20130126 | Germany | Brandenburg | M |
| WE204 | *C. lupus* | scat | 20140206 | Germany | Brandenburg | F |
| WE205 | *C. lupus* | scat | 20131220 | Germany | Brandenburg | U |
| WE207 | *C. lupus* | scat | 20131220 | Germany | Brandenburg | F |
| WE208 | *C. lupus* | scat | 20140210 | Germany | Brandenburg | M |
| WE209 | *C. lupus* | scat | 20131220 | Germany | Brandenburg | U |
| WE212 | *C. lupus* | scat | 20140125 | Germany | Brandenburg | M |
| WE213 | *C. lupus* | scat | 20140122 | Germany | Brandenburg | M |
| WE214 | *C. lupus* | scat | 20140210 | Germany | Brandenburg | U |
| WE215 | *C. lupus* | scat | 20140125 | Germany | Brandenburg | M |
| WE217 | *C. lupus* | scat | 20130125 | Germany | Brandenburg | F |
| WF123 | *C. lupus* | scat | 20140924 | Germany | Sachsen | F |
| WF124 | *C. lupus* | scat | 20140630 | Germany | Sachsen | M |
| WF125 | *C. lupus* | scat | 20141218 | Germany | Sachsen | F |
| WF126 | *C. lupus* | scat | 20141218 | Germany | Sachsen | F |
| WF417 | *C. lupus* | scat | 20150202 | Germany | Sachsen | M |
| WF418 | *C. lupus* | scat | 20150202 | Germany | Sachsen | F |
| WF419 | *C. lupus* | scat | 20150219 | Germany | Sachsen | F |
| WF423 | *C. lupus* | scat | 20150213 | Germany | Sachsen | F |
| WF424 | *C. lupus* | scat | 20150215 | Germany | Sachsen | U |
| WF426 | *C. lupus* | scat | 20150226 | Germany | Sachsen | M |
| WF427 | *C. lupus* | scat | 20150218 | Germany | Sachsen | M |
| WF428 | *C. lupus* | scat | 20150205 | Germany | Sachsen | M |
| WF429 | *C. lupus* | scat | 20150207 | Germany | Sachsen | M |
| WF436 | *C. lupus* | scat | 20150218 | Germany | Sachsen | F |
| WF439 | *C. lupus* | scat | 20150213 | Germany | Sachsen | M |
| FJ133 | *F. s. catus* | hairs | 20151002 | Germany | Bayern | U |
| FJ134 | *F. s. catus* | hairs | 20150302 | Germany | Bayern | M |
| FJ144 | *F. s. silvestris* | hairs | 20150219 | Germany | Nordrhein-Westfalen | U |
| FJ171 | *F. s. silvestris* | hairs | 20150227 | Germany | Rheinland-Pfalz | F |

| | | | | | | |
|---|---|---|---|---|---|---|
| FJ199 | *F. s. silvestris* | hairs | 20150203 | Germany | Bayern | M |
| FJ337 | *F. s. silvestris* | hairs | 20150325 | Germany | Bayern | M |
| FJ412 | *F. s. silvestris* | hairs | 20150314 | Germany | Bayern | U |
| FJ423 | *F. s. catus* | hairs | 20150314 | Germany | Bayern | M |
| FJ477 | *F. s. catus* | hairs | 20151202 | Germany | Baden-Württemberg | F |
| FJ482 | *F. s. silvestris* | hairs | 20150403 | Germany | Hessen | M |
| FJ559 | *F. s. silvestris* | hairs | 20150123 | Germany | Hessen | M |
| FJ601 | *F. s. silvestris* | hairs | 20150113 | Germany | Hessen | F |
| FJ642 | *F. s. catus* | hairs | 20150225 | Germany | Rheinland-Pfalz | F |
| FJ651 | *F. s. silvestris* | hairs | 20150403 | Germany | Hessen | M |
| FJ657 | *F. s. silvestris* | hairs | 20151103 | Germany | Hessen | M |
| FJ699 | *F. s. silvestris* | hairs | 20150222 | Germany | Hessen | M |
| FJ702 | *F. s. silvestris* | hairs | 20150228 | Germany | Hessen | M |
| FJ708 | *F. s. silvestris* | hairs | 20151103 | Germany | Hessen | M |
| FJ777 | *F. s. catus* | hairs | 20150704 | Germany | Saarland | F |
| FJ782 | *F. s. catus* | hairs | 20150319 | Germany | Saarland | F |
| FJ783 | *F. s. silvestris* | hairs | 20150704 | Germany | Saarland | U |
| FJ790 | *F. s. catus* | hairs | 20150319 | Germany | Saarland | F |
| K150465 | *F. s. silvestris* | hairs | 20150603 | Germany | Rheinland-Pfalz | F |
| K150526 | *F. s. silvestris* | hairs | 20151104 | Germany | Thüringen | M |
| K150568 | *F. s. silvestris* | hairs | 20150225 | Germany | Sachsen-Anhalt | F |
| K150571 | *F. s. silvestris* | hairs | 20150225 | Germany | Sachsen-Anhalt | F |
| K150578 | *F. s. catus* | hairs | 20151003 | Germany | Sachsen-Anhalt | M |
| K150584 | *F. s. catus* | hairs | 20121009 | Germany | Niedersachsen | U |
| K150602 | *F. s. catus* | hairs | 20120716 | Germany | Niedersachsen | M |
| K150689 | *F. s. silvestris* | hairs | 20150226 | Germany | Rheinland-Pfalz | M |
| K150706 | *F. s. silvestris* | hairs | 20150503 | Germany | Rheinland-Pfalz | M |
| K150721 | *F. s. silvestris* | hairs | 20150313 | Germany | Rheinland-Pfalz | M |
| K150731 | *F. s. silvestris* | hairs | 20150320 | Germany | Rheinland-Pfalz | F |
| K150828 | *F. s. silvestris* | hairs | 20150903 | Germany | Niedersachsen | M |
| K150835 | *F. s. silvestris* | hairs | 20150903 | Germany | Niedersachsen | F |
| K150848 | *F. s. silvestris* | hairs | 20140729 | Germany | Niedersachsen | U |
| K150858 | *F. s. silvestris* | hairs | 20150403 | Germany | Niedersachsen | F |
| K150861 | *F. s. silvestris* | hairs | 20150327 | Germany | Rheinland-Pfalz | M |
| K150875 | *F. s. silvestris* | hairs | 20150330 | Germany | Rheinland-Pfalz | U |
| K150895 | *F. s. silvestris* | hairs | 20150203 | Germany | Rheinland-Pfalz | F |
| K151063 | *F. s. silvestris* | hairs | 20151103 | Germany | Rheinland-Pfalz | F |
| K151072 | *F. s. silvestris* | hairs | 20150323 | Germany | Rheinland-Pfalz | F |
| K151129 | *F. s. silvestris* | hairs | 20150313 | Germany | Rheinland-Pfalz | M |
| K151200 | *F. s. silvestris* | hairs | 20150326 | Germany | Bayern | U |
| K151264 | *F. s. silvestris* | hairs | 20150406 | Germany | Hessen | M |
| K151294 | *F. s. silvestris* | hairs | 20150519 | Germany | Baden-Württemberg | M |
| K151297 | *F. s. silvestris* | hairs | 20150417 | Germany | Baden-Württemberg | M |
| K151415 | *F. s. silvestris* | hairs | 20150702 | Germany | Hessen | M |
| K151443 | *F. s. silvestris* | hairs | 20150315 | Germany | Hessen | M |
| K151445 | *F. s. silvestris* | hairs | 20150315 | Germany | Hessen | M |
| K151455 | *F. s. silvestris* | hairs | 20150317 | Germany | Niedersachsen | F |
| K151468 | *F. s. silvestris* | hairs | 20150226 | Germany | Niedersachsen | M |
| K151475 | *F. s. catus* | hairs | 20151103 | Germany | Sachsen-Anhalt | F |
| K151811 | *F. s. catus* | hairs | 20150709 | Germany | Bayern | F |
| H1014 | *U. arctos* | hairs | 20080527 | Greece | Central Pindos | M |
| H1057 | *U. arctos* | hairs | 20080527 | Greece | Central Pindos | M |
| H1069 | *U. arctos* | hairs | 20080529 | FYR Macedonia (FYROM) | - | M |
| H1080 | *U. arctos* | hairs | 20080528 | FYR Macedonia (FYROM) | - | F |
| H1087 | *U. arctos* | hairs | 20080529 | FYR Macedonia (FYROM) | - | F |
| H1125 | *U. arctos* | hairs | 20080528 | Greece | Southern Pindos | M |
| H1132 | *U. arctos* | hairs | 20080530 | Greece | Southern Pindos | M |
| H1163 | *U. arctos* | hairs | 20080605 | Greece | Northern Pindos | M |
| H164 | *U. arctos* | hairs | 20050326 | Greece | Central Pindos | M |
| H166 | *U. arctos* | hairs | 20050326 | Greece | Central Pindos | M |
| H169 | *U. arctos* | hairs | 20050326 | Greece | Central Pindos | M |
| H171 | *U. arctos* | hairs | 20050326 | Greece | Central Pindos | M |
| H179 | *U. arctos* | hairs | 20050326 | Greece | Central Pindos | M |
| H218 | *U. arctos* | hairs | 20050430 | Greece | Central Pindos | M |
| H2299 | *U. arctos* | hairs | 20090617 | Greece | Southern Pindos | M |
| H238 | *U. arctos* | hairs | 20050530 | Greece | Central Pindos | M |
| H2454 | *U. arctos* | hairs | 20090625 | Greece | Vitsi - Varnoundas | M |
| H2464 | *U. arctos* | hairs | 20090603 | FYR Macedonia (FYROM) | - | M |

| | | | | | | |
|---|---|---|---|---|---|---|
| H254 | *U. arctos* | hairs | 20050530 | Greece | Central Pindos | M |
| H2557 | *U. arctos* | hairs | 20090730 | Greece | Vitsi - Varnoundas | M |
| H2592 | *U. arctos* | hairs | 20090617 | Serbia | - | M |
| H2595 | *U. arctos* | hairs | 20090617 | Serbia | - | F |
| H2630 | *U. arctos* | hairs | 20090727 | Serbia | - | F |
| H2669 | *U. arctos* | hairs | 20090818 | Greece | Vitsi - Varnoundas | M |
| H2712 | *U. arctos* | hairs | 20090929 | Greece | Central Pindos | M |
| H2716 | *U. arctos* | hairs | - | Albania | - | F |
| H2719 | *U. arctos* | hairs | 20090822 | FYR Macedonia (FYROM) | - | F |
| H2750 | *U. arctos* | hairs | 20091028 | Greece | Central Pindos | F |
| H2767 | *U. arctos* | hairs | 20090824 | Serbia | - | F |
| H279 | *U. arctos* | hairs | 20050530 | Greece | Central Pindos | M |
| H2867 | *U. arctos* | hairs | 20100423 | Greece | Vitsi - Varnoundas | M |
| H3073 | *U. arctos* | hairs | 20100526 | Greece | Vitsi - Varnoundas | M |
| H3099 | *U. arctos* | hairs | 20100526 | Greece | Vitsi - Varnoundas | M |
| H3260 | *U. arctos* | hairs | 20100623 | Greece | Southern Pindos | M |
| H3333 | *U. arctos* | hairs | 20100629 | Greece | Vitsi - Varnoundas | M |
| H3365 | *U. arctos* | hairs | 20100719 | Greece | Vitsi - Varnoundas | M |
| H3390 | *U. arctos* | hairs | 20100720 | Greece | Vitsi - Varnoundas | M |
| H3489 | *U. arctos* | hairs | 20100902 | Greece | Vitsi - Varnoundas | M |
| H3526 | *U. arctos* | hairs | 20100923 | Greece | Mount Voras | F |
| H356 | *U. arctos* | hairs | 20050730 | Greece | Central Pindos | M |
| H435 | *U. arctos* | hairs | 20060330 | Greece | Central Pindos | M |
| H45 | *U. arctos* | hairs | 20030613 | - | - | M |
| H495 | *U. arctos* | hairs | 20060821 | - | - | F |
| H528 | *U. arctos* | hairs | 20070821 | - | - | M |
| H549 | *U. arctos* | hairs | 20071001 | Greece | Vitsi - Varnoundas | F |
| H561 | *U. arctos* | hairs | 20071026 | Greece | Central Pindos | F |
| H589 | *U. arctos* | hairs | 20071114 | Greece | Central Pindos | F |
| H598 | *U. arctos* | hairs | 20071114 | Greece | Central Pindos | M |
| H604 | *U. arctos* | hairs | 20071203 | Greece | Central Pindos | M |
| H610 | *U. arctos* | hairs | 20071203 | Greece | Central Pindos | F |
| H622 | *U. arctos* | hairs | 20071106 | Greece | Southern Pindos | M |
| H649 | *U. arctos* | hairs | 20071005 | Greece | Northern Pindos | M |
| H663 | *U. arctos* | hairs | 20071031 | Greece | Northern Pindos | M |
| H674 | *U. arctos* | hairs | 20071031 | Greece | Northern Pindos | F |
| H678 | *U. arctos* | hairs | 20071031 | Greece | Northern Pindos | M |
| H702 | *U. arctos* | hairs | 20071205 | Greece | Northern Pindos | F |
| H771 | *U. arctos* | hairs | 20080225 | Greece | Central Pindos | M |
| H787 | *U. arctos* | hairs | 20080328 | Greece | Central Pindos | M |
| H794 | *U. arctos* | hairs | 20080402 | Greece | Northern Pindos | M |
| H805 | *U. arctos* | hairs | 20080402 | Greece | Northern Pindos | M |
| H813 | *U. arctos* | hairs | 20080403 | Greece | Northern Pindos | M |
| H819 | *U. arctos* | hairs | 20080403 | Greece | Northern Pindos | M |
| H836 | *U. arctos* | hairs | 20080404 | Greece | Southern Pindos | M |
| H847 | *U. arctos* | hairs | 20080423 | Greece | Vitsi - Varnoundas | M |
| H851 | *U. arctos* | hairs | 20080424 | - | - | M |
| H871 | *U. arctos* | hairs | 20080423 | Greece | Vitsi - Varnoundas | M |
| H877 | *U. arctos* | hairs | 20080425 | Greece | Central Pindos | M |
| H919 | *U. arctos* | hairs | 20080427 | Greece | Central Pindos | M |
| H939 | *U. arctos* | hairs | 20080502 | Albania | - | F |
| H953 | *U. arctos* | hairs | 20080509 | Greece | Northern Pindos | M |
| H955 | *U. arctos* | hairs | 20080509 | Greece | Northern Pindos | M |
| H980 | *U. arctos* | hairs | 20080508 | Greece | Northern Pindos | M |

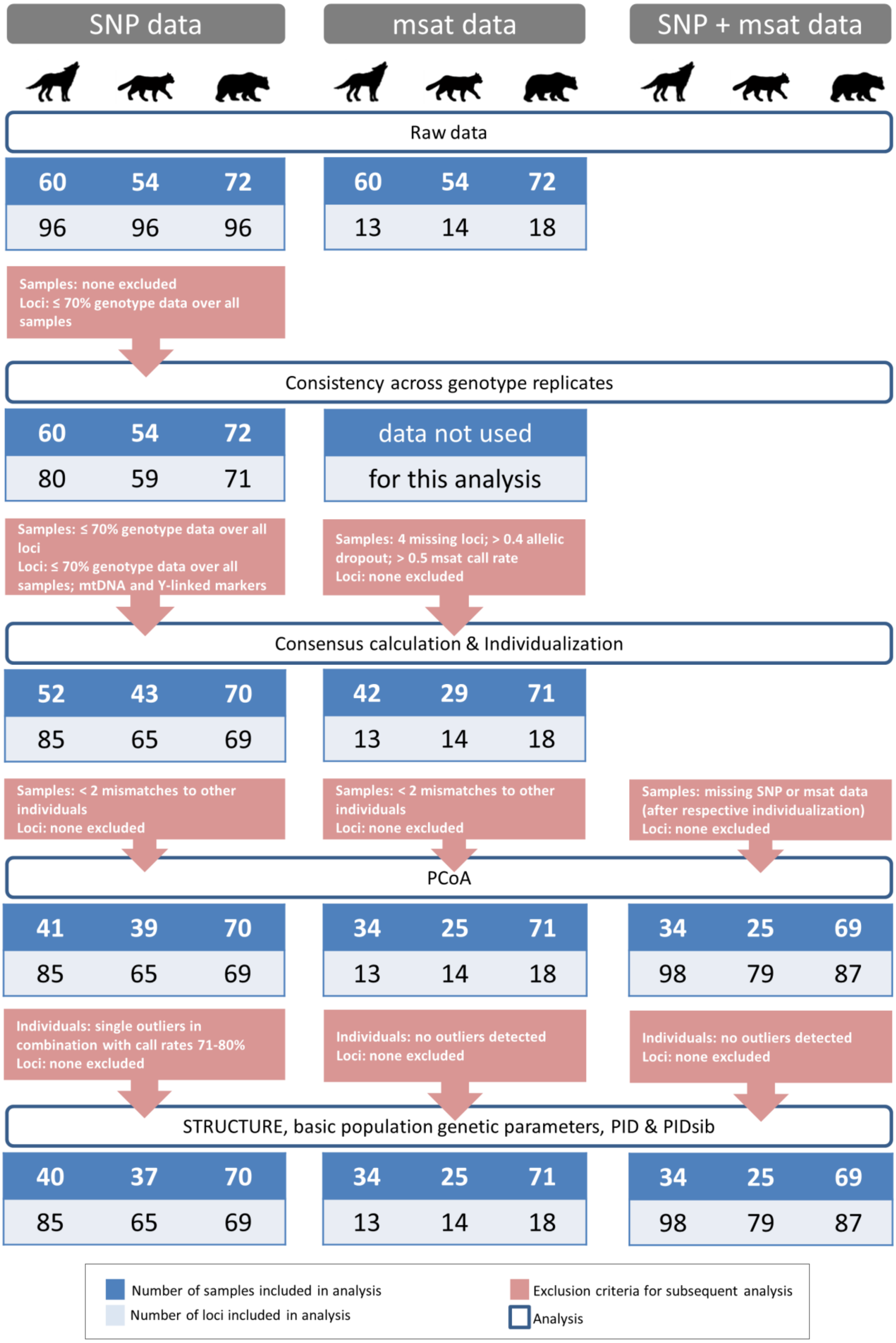


**Supplementary Figure S1** Overview of numbers of samples and loci included in each analysis based on quality criteria.

**Supplementary Table S3** Overview of individuals that were represented by multiple samples in SNP and microsatellite data sets. One mismatch at one locus was accepted to consider two genotypes as belonging to the same individual. Note that brown bear samples had been individualized using microsatellites in the course of a previous study and consequently no matching genotypes were found in this study. *n*, number of samples; f.a., sample failed in microsatellite amplification; NA, matching not available due to failed microsatellite amplification of one of the samples.

| **SNPs** | | | | | | |
|---|---|---|---|---|---|---|
| Species | Individual ID | *n* | sample ID | matches | mismatches | missing loci |
| Grey wolves | A | 2 | WE207, WE217 | 85 | 0 | 0 |
| (*n* loci = 85) | B | 3 | WE170, WE173, WE175 | 84 | 1 | 0 |
| | C | 4 | WE171, WE201, WE208, WE213 | 85 | 0 | 0 |
| | D | 3 | WE126, WE212, WE215 | 85 | 0 | 0 |
| | E | 2 | WF423, WF424 | 82 | 1 | 2 |
| | F | 2 | WF428, WF429 | 83 | 0 | 2 |
| | G | 2 | WE167, WE161 | 85 | 0 | 0 |
| European | H | 2 | K150052, K150075 | 64 | 0 | 1 |
| wildcats | I | 2 | K150689, K150706 | 63 | 1 | 1 |
| (*n* loci = 65) | J | 2 | K151294, K151297 | 65 | 0 | 0 |
| | K | 2 | K151415, K151443 | 65 | 0 | 0 |
| **Microsatellites** | | | | | | |
| Grey wolves | A | 1 | WE207[f.a.], WE217 | NA | NA | NA |
| (*n* loci = 13) | B | 2 | WE170, WE173, WE175[f.a.] | 11 | 1 | 1 |
| | C | 4 | WE171, WE201, WE208, WE213 | 10 | 1 | 2 |
| | D | 3 | WE126, WE212, WE215 | 12 | 1 | 0 |
| | E | 2 | WF423, WF424 | 12 | 1 | 0 |
| | F | 2 | WF428, WF429 | 13 | 0 | 0 |
| | G | 1 | WE167, WE161[f.a.] | NA | NA | NA |
| European | H | 2 | K150052, K150075 | 14 | 0 | 0 |
| wildcats | I | 2 | K150689, K150706 | 14 | 0 | 0 |
| (*n* loci = 14) | J | 2 | K151294, K151297 | 13 | 0 | 1 |
| | K | 2 | K151415, K151443 | 14 | 0 | 0 |

**Supplementary Table S4** Genetic variability in grey wolves, European wildcats and brown bears for SNPs and microsatellites. *n*, sample size; *Na*, mean number of different alleles over loci; $H_O$, observed heterozygosity; $H_E$; expected heterozygosity; ML, number of monomorphic loci. For sample groups with $n \leq 5$ no calculations were performed (5 grey wolves, 2 potential wildcat hybrids, 16 brown bears).

| **SNPs** | | | | | | |
|---|---|---|---|---|---|---|
| Species | Groups | *n* | *Na* | $H_O$ | $H_E$ | ML |
| Grey wolf | Germany, Brandenburg | 9 | 1.79 ± 0.05 | 0.32 ± 0.03 | 0.28 ± 0.02 | 18 |
| | Germany, Sachsen | 17 | 1.81 ± 0.04 | 0.30 ± 0.03 | 0.27 ± 0.02 | 16 |
| | Germany, Sachsen-Anhalt | 9 | 1.84 ± 0.04 | 0.34 ± 0.03 | 0.28 ± 0.02 | 14 |
| | **Total** | 40 | 1.91 ± 0.03 | 0.31 ± 0.02 | 0.29 ± 0.02 | 8 |
| European wildcat[a] | Germany, Domestic cat | 7 | 1.77 ± 0.05 | 0.21 ± 0.03 | 0.24 ± 0.02 | 15 |
| | Germany, Wildcat | 28 | 1.88 ± 0.04 | 0.11 ± 0.02 | 0.12 ± 0.02 | 8 |
| | **Total** | 37 | 2.00 ± 0.00 | 0.16 ± 0.01 | 0.35 ± 0.01 | 0 |
| Brown bear | Greece, Central Pindos | 23 | 1.81 ± 0.05 | 0.26 ± 0.02 | 0.29 ± 0.02 | 13 |
| | Greece, Northern Pindos | 13 | 1.80 ± 0.05 | 0.27 ± 0.03 | 0.29 ± 0.03 | 14 |
| | Greece, Southern Pindos | 6 | 1.75 ± 0.05 | 0.26 ± 0.03 | 0.27 ± 0.03 | 17 |
| | Greece, Vitsi - Varnoundas | 13 | 1.84 ± 0.04 | 0.28 ± 0.02 | 0.32 ± 0.02 | 11 |
| | **Total** | 70 | 1.90 ± 0.04 | 0.28 ± 0.02 | 0.31 ± 0.02 | 7 |
| **Microsatellites** | | | | | | |
| Grey wolf | Germany, Brandenburg | 7 | 3.31 ± 0.24 | 0.51 ± 0.06 | 0.54 ± 0.05 | 0 |
| | Germany, Sachsen | 15 | 3.85 ± 0.36 | 0.62 ± 0.06 | 0.53 ± 0.05 | 0 |
| | Germany, Sachsen-Anhalt | 8 | 2.92 ± 0.29 | 0.46 ± 0.07 | 0.49 ± 0.07 | 1 |
| | **Total** | 34 | 4.46 ± 0.39 | 0.54 ± 0.05 | 0.56 ± 0.04 | 0 |
| European wildcat | Germany, Domestic cat | 6 | 5.29 ± 0.38 | 0.63 ± 0.04 | 0.77 ± 0.04 | 0 |
| | Germany, Wildcat | 18 | 6.00 ± 0.50 | 0.64 ± 0.04 | 0.71 ± 0.03 | 0 |
| | **Total** | 25 | 7.86 ± 0.74 | 0.65 ± 0.04 | 0.77 ± 0.02 | 0 |
| Brown bear | Greece, Central Pindos | 23 | 4.50 ± 0.22 | 0.67 ± 0.03 | 0.66 ± 0.02 | 0 |
| | Greece, Northern Pindos | 13 | 4.50 ± 0.22 | 0.68 ± 0.04 | 0.67 ± 0.03 | 0 |
| | Greece, Southern Pindos | 6 | 3.83 ± 0.25 | 0.71 ± 0.08 | 0.71 ± 0.03 | 0 |
| | Greece, Vitsi - Varnoundas | 13 | 4.95 ± 0.24 | 0.74 ± 0.04 | 0.70 ± 0.02 | 0 |
| | **Total** | 71 | 7.06 ± 0.42 | 0.70 ± 0.03 | 0.72 ± 0.02 | 0 |

[a] Note that the SNP panel for wildcats was designed to detect hybridization between wildcats and domestic cats.

**Supplementary Table S5** Pairwise $F_{ST}$ values for grey wolves with microsatellite data (above the diagonal, $n$ = 30 samples and 13 loci) and SNP data (below the diagonal, $n$ = 35 samples and 85 loci). All samples were collected in Germany. Only groups with $n$ > 5 were considered (5 wolves from 2 locations excluded). Probability values were based on 999 permutations; *$p \leq 0.05$; **$p \leq 0.01$; ***$p \leq 0.001$.

| | Brandenburg | Sachsen | Sachsen-Anhalt |
|---|---|---|---|
| **Brandenburg** | - | 0.08** | 0.05* |
| **Sachsen** | 0.08** | - | 0.10*** |
| **Sachsen-Anhalt** | 0.06* | 0.10*** | - |

**Supplementary Table S6** Pairwise $F_{ST}$ values for European wildcats and domestic cats with microsatellite data (above the diagonal, $n$ = 24 samples and 14 loci) and SNP data (below the diagonal, $n$ = 35 samples and 65 loci). Note that the SNP panel we used here was designed to detect hybridization of wildcats and domestic cats (that is, maximize differentiation). All samples were collected in Germany. Potential hybrids (based on SNP data) were excluded from these analyses ($n$ = 1 for msats, $n$ = 2 for SNPs). Probability values were based on 999 permutations; ***$p \leq 0.001$.

| | Domestic cat | Wildcat |
|---|---|---|
| **Domestic cat** | - | 0.13*** |
| **Wildcat** | 0.79*** | - |

**Supplementary Table S7** Pairwise $F_{ST}$ values for brown bears with microsatellite data (above the diagonal, $n$ = 55 samples and 18 loci) and SNP data (below the diagonal, $n$ = 55 samples and 69 loci). All samples were collected in Greece. Only groups with $n$ > 5 were considered (16 bears from 5 locations excluded). Probability values were based on 999 permutations; n.s., not significant; *$p \leq 0.05$; **$p \leq 0.01$; ***$p \leq 0.001$. Negative values were converted to zero.

| | Central Pindos | Northern Pindos | Southern Pindos | Vitsi - Varnoundas |
|---|---|---|---|---|
| **Central Pindos** | - | 0.03** | 0.03* | 0.05*** |
| **Northern Pindos** | 0.04** | - | $0.02^{n.s.}$ | 0.03* |
| **Southern Pindos** | $0.00^{n.s.}$ | $0.03^{n.s.}$ | - | 0.04* |
| **Vitsi – Varnoundas** | 0.06*** | 0.03* | 0.04* | - |

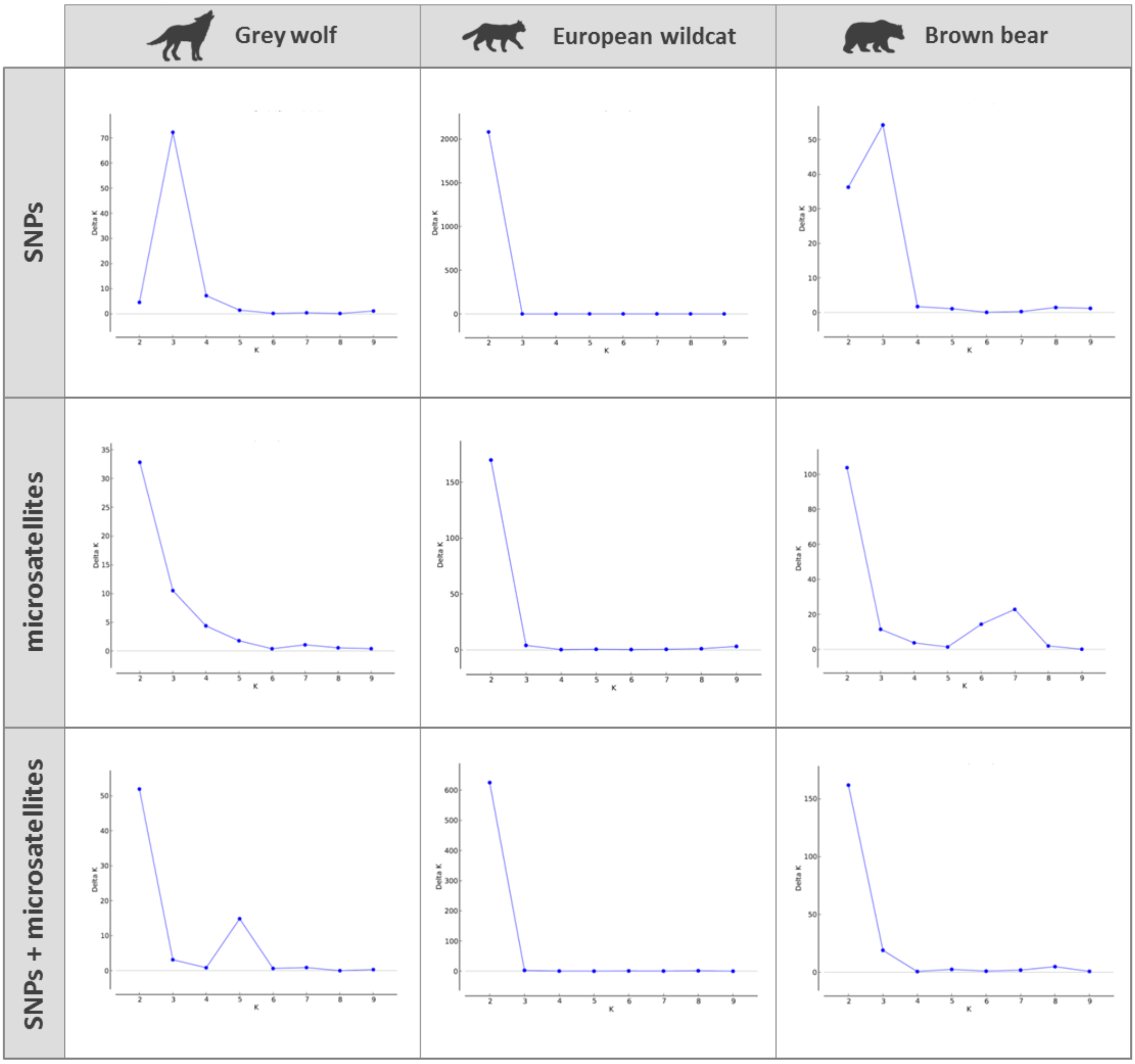


**Supplementary Figure S2** Delta *K* for STRUCTURE runs corresponding to Supplementary Figure S3.

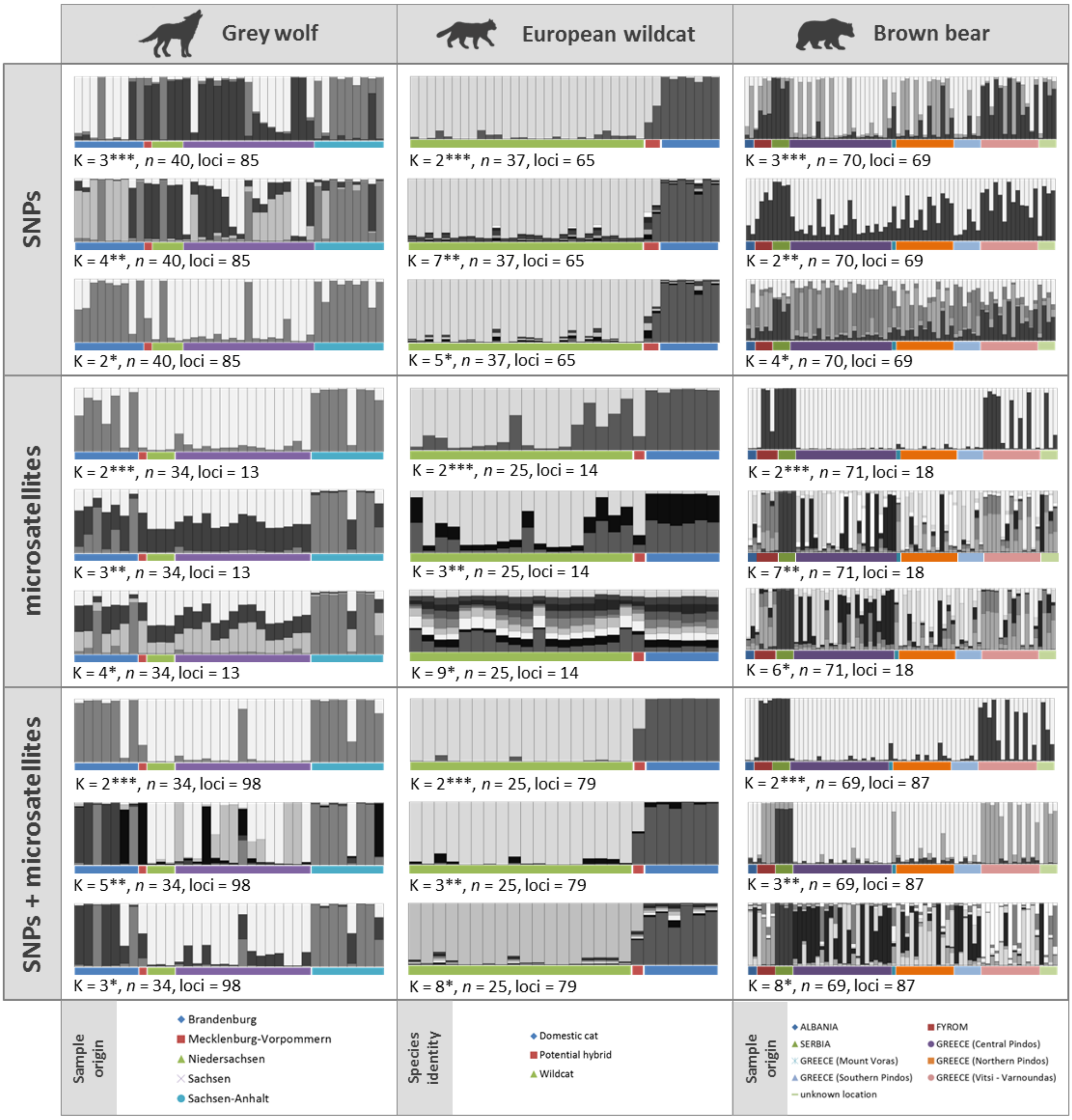


**Supplementary Figure S3** STRUCTURE plots showing results for the most likely *K* (***), second most likely *K* (**) and third most likely *K* (*) as calculated with the Evanno method based on SNP and microsatellite genotypes and their combination. Colour-coded bars below the STRUCTURE plots correspond to the sample groupings based on sampling region (grey wolves, brown bears) or species identification (wildcats or domestic cats, based on SNP data).

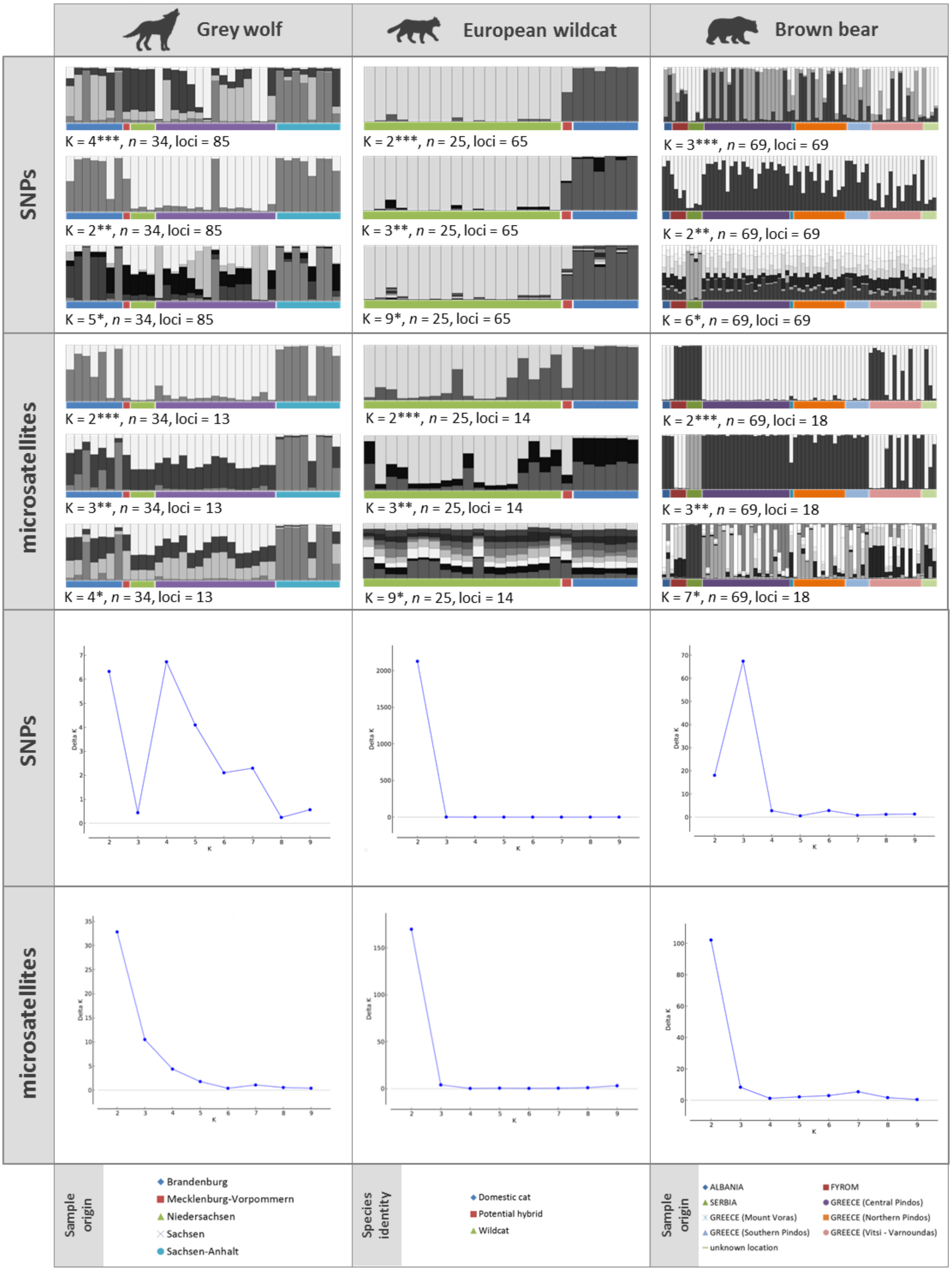


**Supplementary Figure S4** STRUCTURE plots showing results for the most likely *K* (***), second most likely *K* (**) and third most likely *K* (*) (upper panels) as calculated with the Evanno method (lower panels, respectively) based on SNP and microsatellite data sets. Colour-coded bars below the STRUCTURE plots correspond to the sample groupings based on sampling region (grey wolves, brown bears) or species identification (wildcats and domestic cats, based on SNP data).

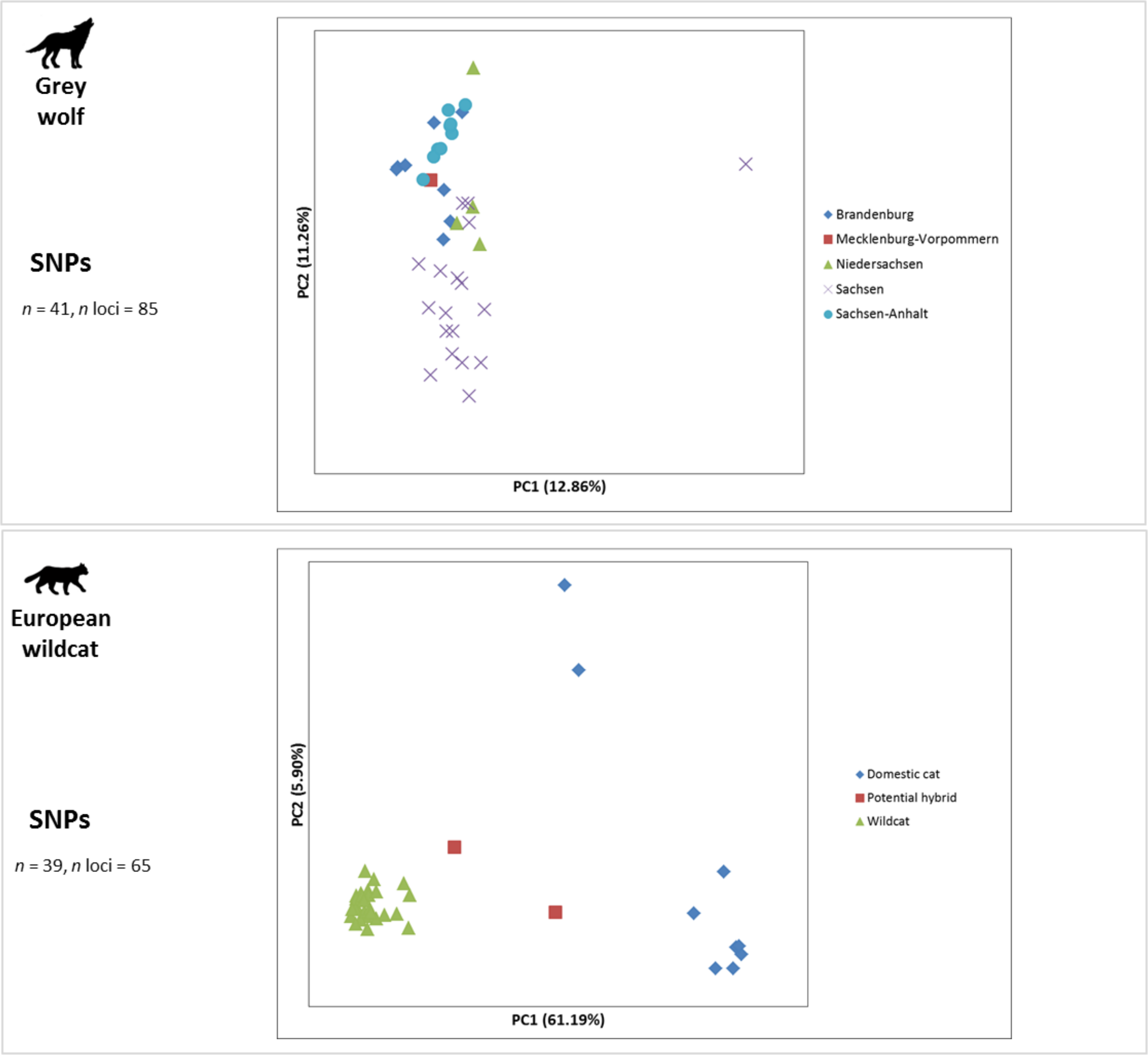


**Supplementary Figure S5** PCoA for wolves and wildcats showing outliers (SNP data, original data set). Further examination of these samples indicated low SNP call rates (wolf 71%; wildcats 77%, 78%). These samples were removed from the figure in the main text, Figure 3, and from further analyses.

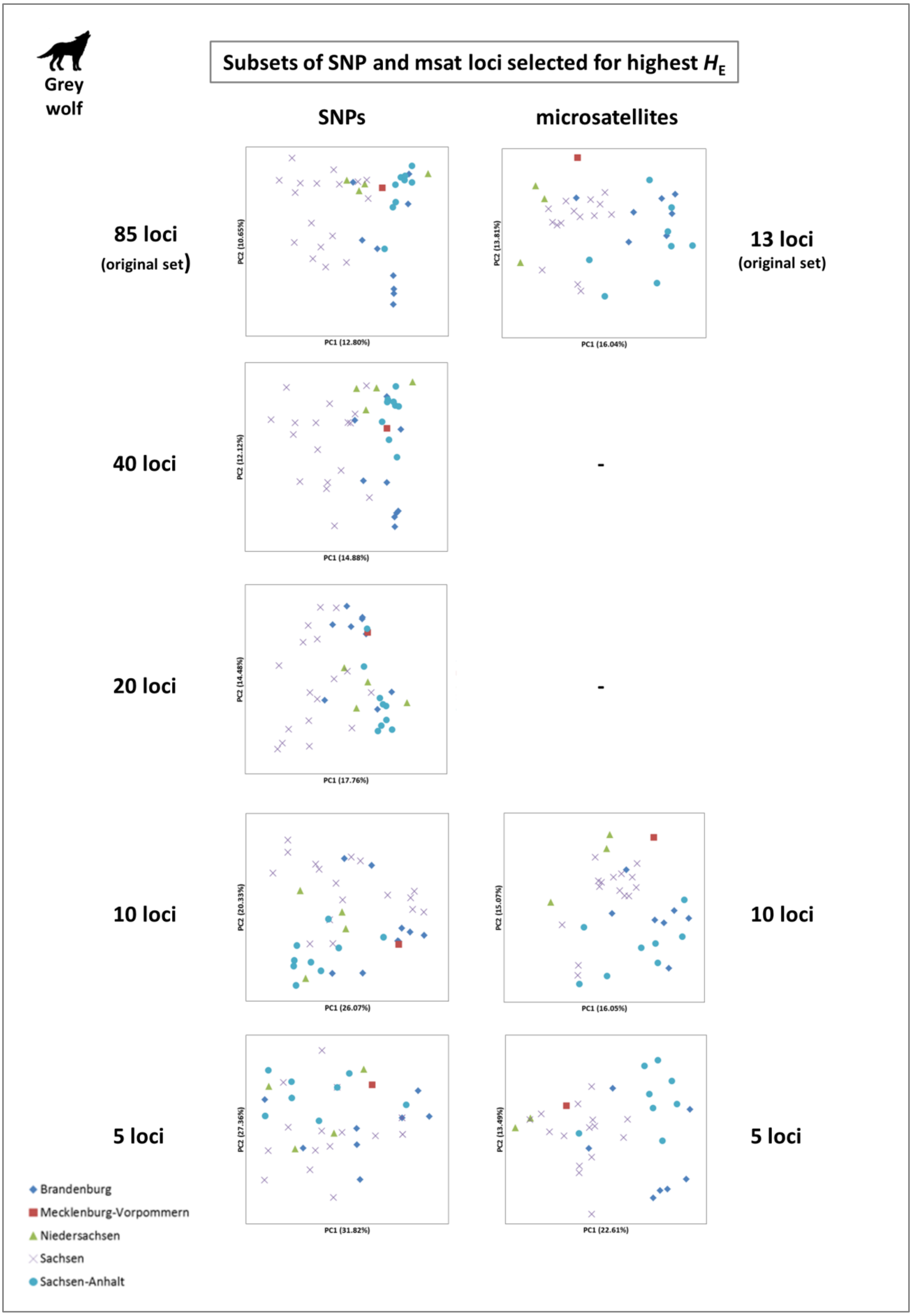


**Supplementary Figure S6** PCoA analyses for subsets of SNP and microsatellite markers used in this study to genotype grey wolves. Each point represents an individual's genotype, colour-coded to its sampling region. Subsets of loci were selected based on highest heterozygosity ($H_E$) for each locus.

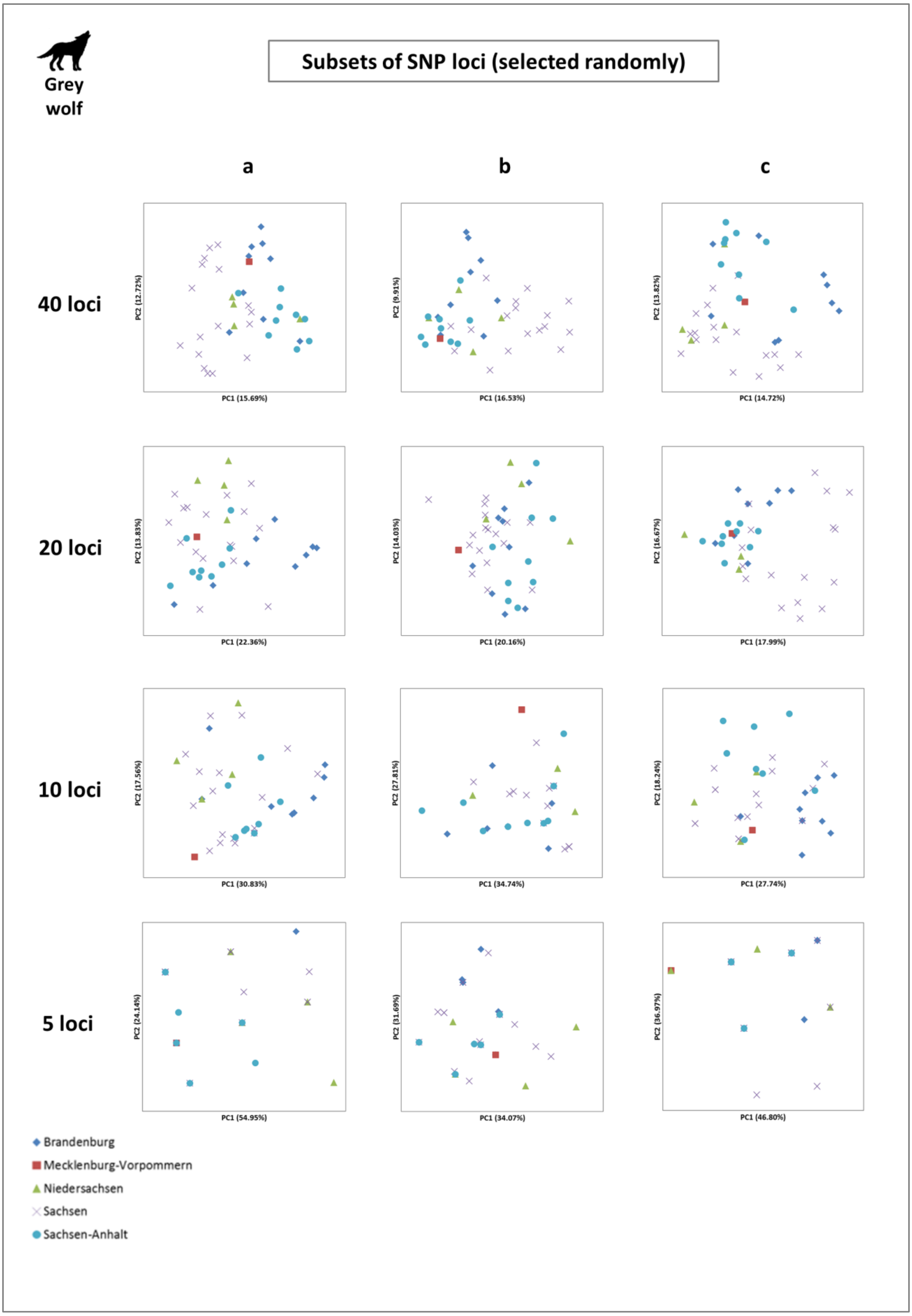


**Supplementary Figure S7** PCoA analyses for subsets of SNP markers used in this study to genotype grey wolves. Each point represents an individual's genotype, colour-coded to its sampling region. Subsets of loci were selected randomly; three times each case (a, b, c).

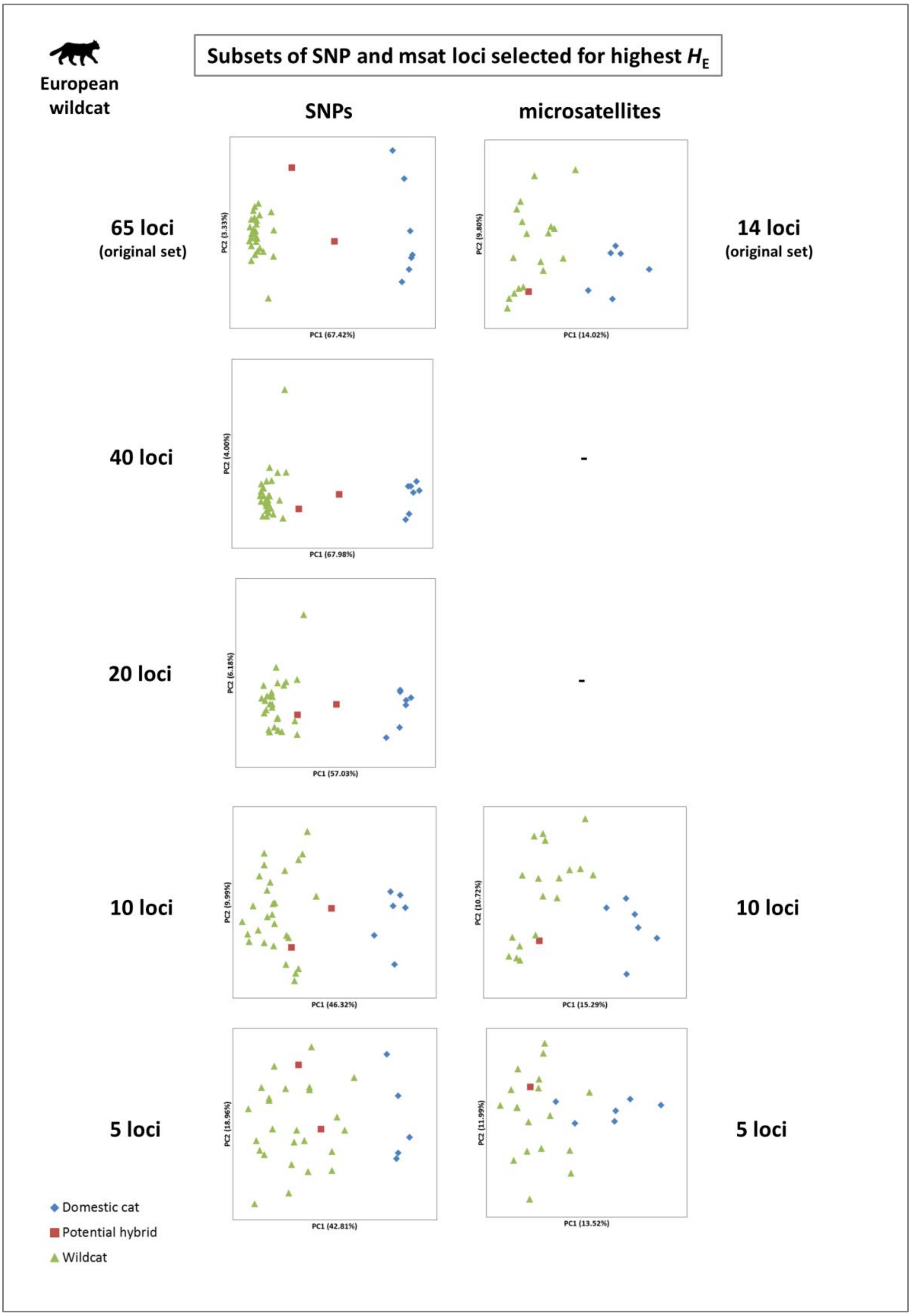


**Supplementary Figure S8** PCoA analyses for subsets of SNP and microsatellite markers used in this study to genotype European wildcats, domestic cats and hybrids. Each point represents an individual's genotype, colour-coded to species identity. Subsets of loci were selected based on highest heterozygosity ($H_E$) for each locus.

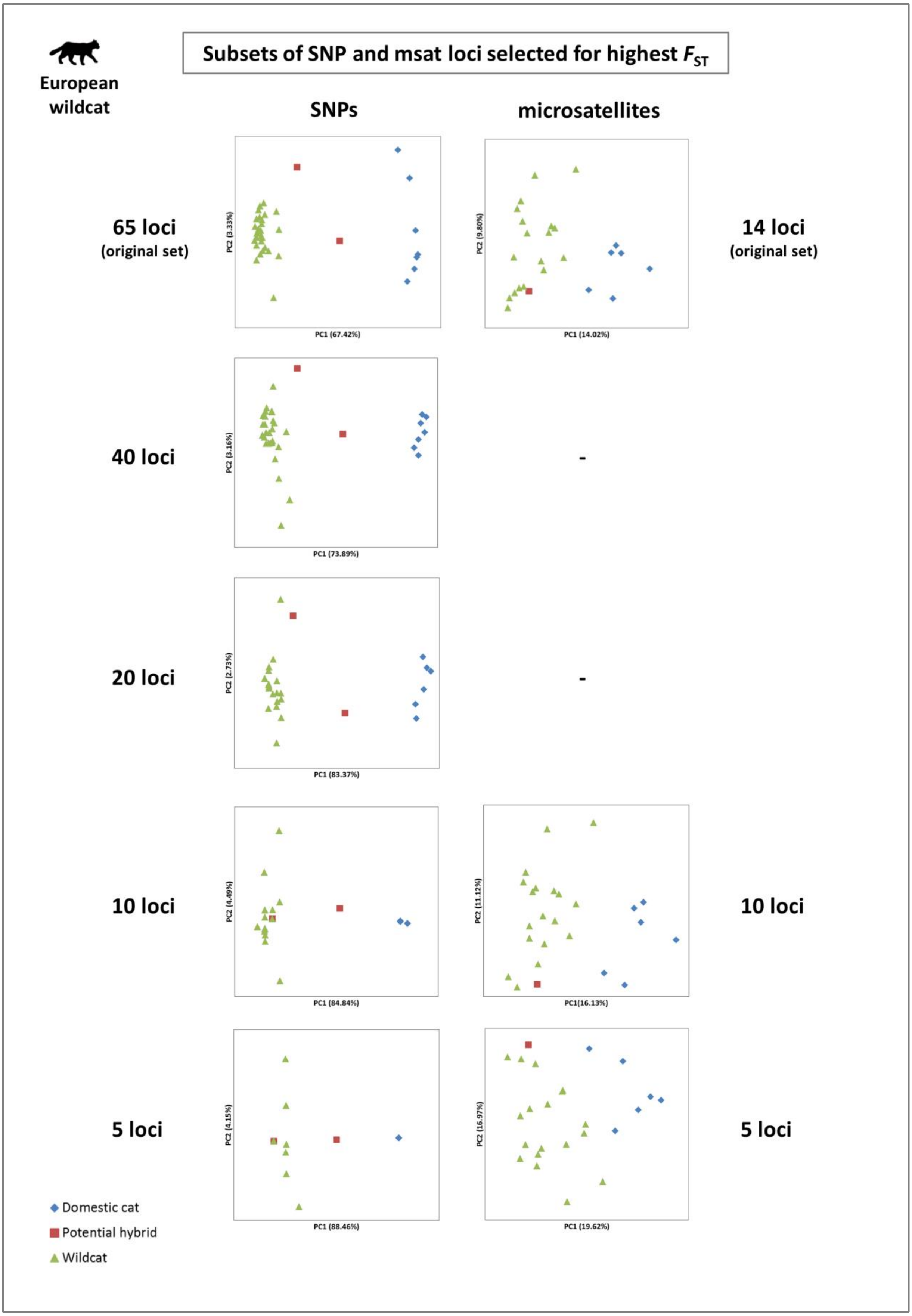


**Supplementary Figure S9** PCoA analyses for subsets of SNP and microsatellite markers used in this study to genotype European wildcats, domestic cats and hybrids. Each point represents an individual's genotype, colour-coded to species identity. Subsets of loci were selected based on highest $F_{ST}$ for each locus.

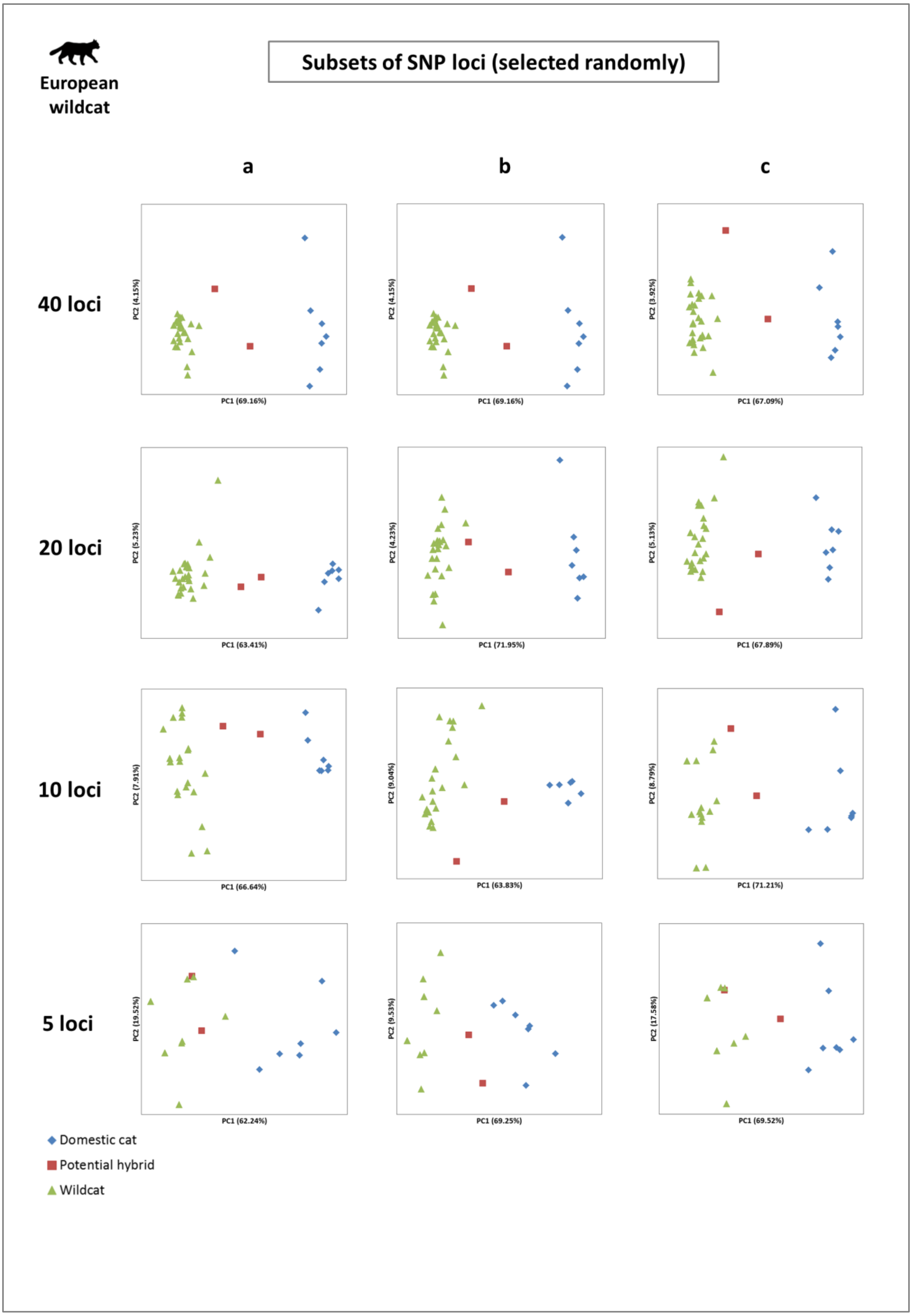


**Supplementary Figure S10** PCoA analyses for subsets of SNP markers used in this study to genotype European wildcats, domestic cats and hybrids. Each point represents an individual's genotype, colour-coded to species identity. Subsets of loci were selected randomly; three times each case (a, b, c).

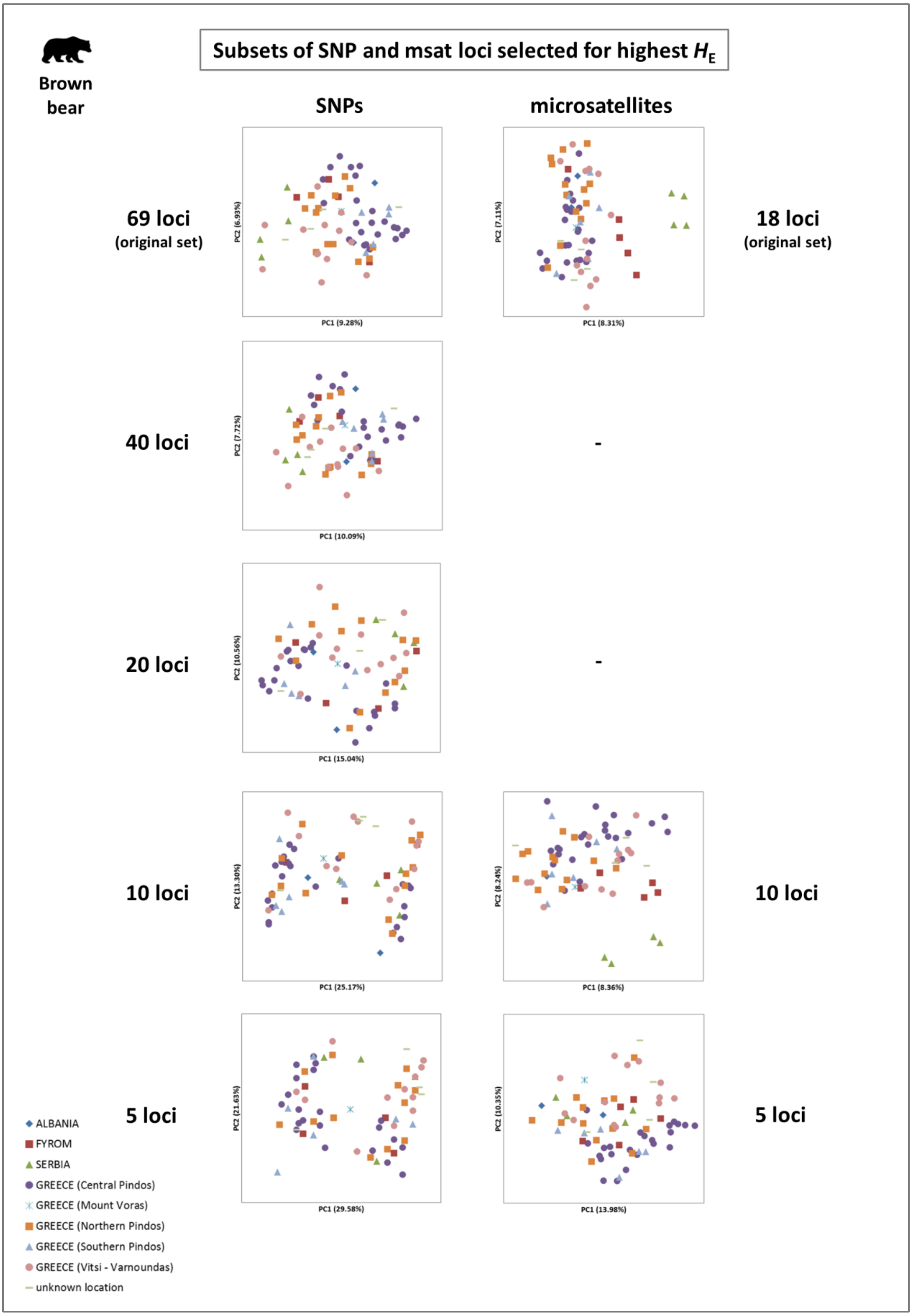


**Supplementary Figure S11** PCoA analyses for subsets of SNP and microsatellite markers used in this study to genotype brown bears. Each point represents an individual's genotype, colour-coded to its sampling region. Subsets of loci were selected based on highest heterozygosity ($H_E$) for each locus.

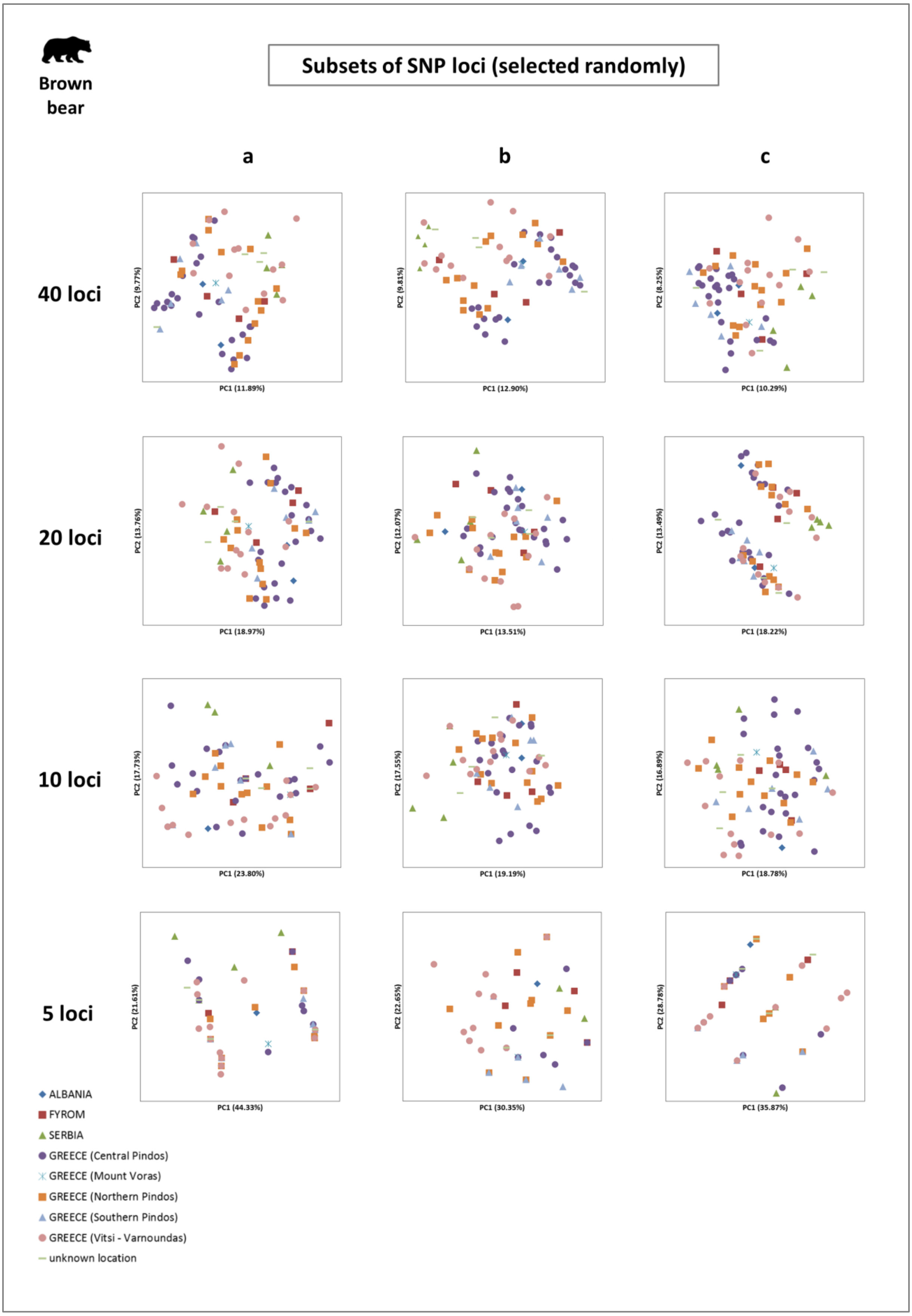


**Supplementary Figure S12** PCoA analyses for subsets of SNP markers used in this study to genotype brown bears. Each point represents an individual's genotype, colour-coded to its sampling region. Subsets of loci were selected randomly; three times each case (a, b, c).

## References


1. Kearse, M. *et al.* Geneious Basic: an integrated and extendable desktop software platform for the organization and analysis of sequence data. *Bioinformatics* **28,** 1647–1649 (2012).
2. Eckert, I., Suchentrunk, F., Markov, G. & Hartl, G. B. Genetic diversity and integrity of German wildcat (*Felis silvestris*) populations as revealed by microsatellites, allozymes, and mitochondrial DNA sequences. *Mamm. Biol.* **75,** 160–174 (2010).
3. Fumagalli, L., Taberlet, P., Favre, L. & Hausser, J. Origin and evolution of homologous repeated sequences in the mitochondrial DNA control region of shrews. *Mol. Biol. Evol.* **13,** 31–46 (1996).
4. Taberlet, P. & Bouvet, J. Mitochondrial DNA polymorphism, phylogeography, and conservation genetics of the brown bear *Ursus arctos* in Europe. *Proc. R. Soc. B* **255,** 195–200 (1994).
5. Karamanlidis, A. A., Hernando, M. d. G., Krambokoukis, L. & Gimenez, O. Evidence of a large carnivore population recovery. Counting bears in Greece. *J. Nat. Conserv.* **27,** 10–17 (2015).
6. Harms, V., Nowak, C., Carl, S. & Muñoz-Fuentes, V. Experimental evaluation of genetic predator identification from saliva traces on wildlife kills. *J. Mammal.* **96,** 138–143 (2015).
7. Steyer, K., Simon, O., Kraus, Robert H. S., Haase, P. & Nowak, C. Hair trapping with valerian-treated lure sticks as a tool for genetic wildcat monitoring in low-density habitats. *Eur. J. Wildl. Res.* **59,** 39–46 (2013).
8. Karamanlidis, A. A. *et al.* Genetic diversity, structure, and size of an endangered brown bear population threatened by highway construction in the Pindos Mountains, Greece. *Eur. J. Wildl. Res.* **58,** 511–522 (2012).
